# Two-tiered mutualism improves survival and competitiveness of cross-feeding soil bacteria

**DOI:** 10.1038/s41396-023-01519-5

**Published:** 2023-09-22

**Authors:** Zhan-Biao Ge, Zhi-Qiang Zhai, Wan-Ying Xie, Jun Dai, Ke Huang, David R. Johnson, Fang-Jie Zhao, Peng Wang

**Affiliations:** 1 Jiangsu Collaborative Innovation Center for Solid Organic Waste Resource Utilization, College of Resources and Environmental Sciences, Nanjing Agricultural University, Nanjing, 210095 China; 2 Centre for Agriculture and Health, Academy for Advanced Interdisciplinary Studies, Nanjing Agricultural University, Nanjing, 210095 China; 3 Department of Environmental Microbiology, Swiss Federal Institute of Aquatic Science and Technology (Eawag), 8600 Dübendorf, Switzerland; 4 Institute of Ecology and Evolution, University of Bern, 3012 Bern, Switzerland

**Keywords:** Soil microbiology, Microbial ecology

## Abstract

Metabolic cross-feeding is a pervasive microbial interaction type that affects community stability and functioning and directs carbon and energy flows. The mechanisms that underlie these interactions and their association with metal/metalloid biogeochemistry, however, remain poorly understood. Here, we identified two soil bacteria, *Bacillus* sp. BP-3 and *Delftia* sp. DT-2, that engage in a two-tiered mutualism. Strain BP-3 has low utilization ability of pyruvic acid while strain DT-2 lacks hexokinase, lacks a phosphotransferase system, and is defective in glucose utilization. When strain BP-3 is grown in isolation with glucose, it releases pyruvic acid to the environment resulting in acidification and eventual self-killing. However, when strain BP-3 is grown together with strain DT-2, strain DT-2 utilizes the released pyruvic acid to meet its energy requirements, consequently rescuing strain BP-3 from pyruvic acid-induced growth inhibition. The two bacteria further enhance their collective competitiveness against other microbes by using arsenic as a weapon. Strain DT-2 reduces relatively non-toxic methylarsenate [MAs(V)] to highly toxic methylarsenite [MAs(III)], which kills or suppresses competitors, while strain BP-3 detoxifies MAs(III) by methylation to non-toxic dimethylarsenate [DMAs(V)]. These two arsenic transformations are enhanced when strains DT-2 and BP-3 are grown together. The two strains, along with their close relatives, widely co-occur in soils and their abundances increase with the soil arsenic concentration. Our results reveal that these bacterial types employ a two-tiered mutualism to ensure their collective metabolic activity and maintain their ecological competitive against other soil microbes. These findings shed light on the intricateness of bacterial interactions and their roles in ecosystem functioning.

## Introduction

Soil is a dynamic ecosystem where changes in microbial communities, species interactions, and the environment are continuously feeding back on each other. Metabolic interactions between species, such as cross-feeding and the detoxification of metabolic waste, are important determinants of microbial survival and growth with consequent effects on community structure, ecosystem functioning [1, 2], and the maintenance of biodiversity [3, 4]. Cross-feeding of metabolic byproducts, such as ethanol and acetic acid, is critical for the diversity of cellulose-degrading communities [5]. Furthermore, pervasive cross-feeding influences global biogeochemical cycles [6, 7]. Nutrient limitations often promote cross-feeding in natural ecosystems, where cross-feeding microbes acquire essential nutrients from other microbes and thus expand their ecological niches [8–10]. However, despite its importance, the mechanisms underlying cross-feeding and their significance in microbial ecology are still not well understood.

Cross-feeding interactions between bacteria can be mediated through transfer of metabolites, such as organic acids, amino acids, sugars, vitamins, and inorganic elements [2, 7–9, 11]. These material transfers can be unidirectional, bidirectional (metabolites are reciprocally exchanged between two bacteria), or even multidirectional, where metabolites are transferred and utilized between multiple microbes to form an interaction network [12–14]. Organic acids, produced by most bacteria when consuming sugars as a carbon source, are critical intermediates in global carbon cycles [13, 15]. Organic acids can be nutrients at low concentrations in the environment but can become inhibitory at high concentrations [14–17]. Differences in organic acid utilization capabilities between microbes may mediate cross-feeding.

In addition to cross-feeding interactions, antagonistic interactions are also common in microbial communities [18]. Antimicrobials produced by microbes can be used as competitive weapons in microbial warfare, which can shape microbial communities by modulating the types of interactions that occur among bacteria [19–21]. For example, some bacteria produce highly toxic methylarsenite [MAs(III)], which has antimicrobial activity [22–26]. MAs(III) is produced either through biomethylation of arsenite [As(III)] [27, 28] or by reduction of relatively non-toxic methylarsenate [MAs(V)] [23, 24, 28, 29]. The production of MAs(III) is a function that likely evolved in the ancient Earth before the great oxidation event [25]. Arsenite [As(III)], a major inorganic arsenic species in the environment, can be methylated by S-adenosylmethionine methyltransferase (encoded by *arsM* in bacteria) [30] in three steps to MAs(III), dimethylarsenite [DMAs(III)], and trimethylarsenicals [TMAs(III)] [31–34]. Trivalent methylarsenicals are readily abiotically oxidized to pentavalent species by oxygen [25]. The primary function of *arsM* is to confer resistance through transformation of As(III) or MAs(III) [30, 35, 36]. Some bacteria, such as *Clostridium* sp. BXM and *Streptomyces* sp. GSRB54, methylate As(III) to MAs(III) as the significant product [37, 38]. In addition to the general biological sources of MAs(V), MAs(V) has been widely used as a herbicide and defoliant [39]. All these MAs(V) can be used as substrates to generate MAs(III).

In this study, we isolated two bacterial strains (*Bacillus* sp. BP-3 and *Delftia* sp. DT-2) from arsenic-contaminated soil and investigated the mechanisms underlying their co-occurrence. Using a combination of experiments, we revealed a two-tiered mutualism between the two strains, where a mutualistic cross-feeding interaction reciprocally promotes their growth while an antagonistic interaction enhances the collective competitiveness of the two strains against other microbes. We propose that such a two-tiered mutualism is common in the biosphere.

## Materials and methods

### Bacterial strains and growth media

We used bacterial strains *Delftia* sp. DT-2, *Bacillus* sp. BP-3, *Bacillus* sp. BT-39, *Chryseobacterium* sp. CT-33, and *Paenarthrobacter* sp. PN-5, *Bacillus* sp. B12, *Bacillus* sp. B38, and *Bacillus* sp. B36. We conducted all cross-feeding and arsenical transformation experiments in ST10^−1^ liquid culture medium (Table S1). We performed colony counting on Luria Bertani (LB) solid culture medium (Table S1). We grew all liquid cultures under oxic conditions at 30 °C with continuous mixing at 200 rpm and all solid cultures in ambient air at 30 °C. Unless specifically stated otherwise, all cultures were monocultures.

We used *Escherichia coli* DH5α for plasmid construction and replication and *E. coli* AW3110 DE3, an arsenic-hypersensitive strain lacking the chromosomal *arsRBC* operon, for arsenic resistance assays. For protein expression, we used *E. coli* BL21 DE3. Lastly, we used *E. coli* W3110 as a control for the pyruvate dehydrogenase activity assay. We grew all *E. coli* strains individually under oxic conditions either in LB or M9 minimal salts media at 37 °C with continuous mixing at 200 rpm.

### Strain isolation and identification

We obtained strains DT-2, BP-3, BT-39, CT-33, and PN-5 from soil samples collected in Chenzhou (CZ), Hunan Province, China. To isolate and identify these strains, we mixed soil with ST10^-1^ liquid culture medium containing 2 μM MAs(V) at a ratio of 1:10 (g:mL) and incubated the mixture at 25 °C and 200 rpm for 24 h. We then diluted the enrichment solution, deposited it onto ST10^-1^ agar plates with 2 μM MAs(V), and incubated the plates at 25 °C for 3 days. Next, we picked individual colonies from the plates using inoculating loops. Strains BT-39, CT-33, and PN-5 formed well-isolated colonies, while strains DT-2 and BP-3 formed colonies that were in physical contact with each other and difficult to separate (Fig. S1). We further purified strains DT-2 and BP-3 by repeatedly streaking the colonies until obtaining well-isolated single colonies. To assign taxonomy to each strain, we amplified and sequenced the 16 S rRNA gene sequences using the primer pair 27F–1492 R (Table S2) and constructed a neighbor-joining phylogenetic tree by aligning the resulting sequences with those in the NCBI database using MEGA X (Fig. S2) [40]. We deposited the 16 S rRNA sequences in GenBank under the accession numbers OP247621 (DT-2) and OP247622 (BP-3).

### Co-culture experiments

To investigate interactions between DT-2 and BP-3, CT-33, BT39, or PN-5, we conducted co-culture experiments in ST10^-1^ liquid culture medium for two days. We grew the strains in mono- or co-culture at a 1:1 ratio. We also conducted a control experiment using only ST10^-1^ culture medium without any bacterial inoculation. At the end of the cultivation, we measured the pH of the culture medium. We quantified the growth of the two strains using either real-time quantitative PCR (qPCR) or colony counting. The bacterial culture solution was gradually diluted with PBS buffer (pH 7.2) and subsequently spread onto LB agar plates. The plates were then incubated at 30 °C for 1 to 3 days, after which we enumerated the colonies for each strain. Notably, there is a noticeable differentiation in the colony morphologies of strains DT-2, BP-3, and the other strains employed in this study. To visualize their interactions, we streaked DT-2 and BP-3 along lines that were parallel to or that crossed each other on the same ST10^-1^ agar plate.

### Genome sequencing and qPCR to assess the growth of DT-2 and BP-3

To assess the growth of DT-2 and BP-3 in mono- and co-culture, we first isolated total DNA from the cultures and performed whole-genome sequencing. The draft genome of DT-2 (GenBank accession number: JAQOTI000000000) solely contains the *arsI* gene that encodes for a C−As bond lyase, while the draft genome of BP-3 (GenBank accession number: JAQOTJ000000000) solely contains the *gerM* gene that encodes for a spore germination protein. We then designed qPCR primers to target these two genes (see Table S3) and used the gene copy numbers to quantify the abundances and growth of the corresponding strains [41]. For detailed protocols, please refer to the Supporting Information.

### Supernatant amendment experiments

To investigate the mechanisms by which DT-2 and BP-3 promote each other’s growth, we performed reciprocal supernatant amendment experiments. First, we separately cultivated DT-2 and BP-3 in ST10^–1^ liquid medium for three days, collected the culture supernatants by centrifugation, and filtered the supernatant using a 0.22 μm MCE syringe filters (ASD, China). Next, we mixed 10 mL of the filtered supernatant with 10 mL of fresh ST10^–1^ liquid culture medium, inoculated BP-3 and DT-2 into the medium containing supernatant from the other strain, and incubated the cultures for two days at 30 °C with shaking at 200 rpm. The control treatment consisted of fresh ST10^-1^ liquid culture medium. We measured the optical density at 600 nm (OD_600_) of the cultures at various times after inoculation.

To determine the impact of pH on the growth of BP-3, we adjusted the pH of the filtered supernatant of the DT-2 culture, which had an original pH of approximately 7.9 to 7.0 by adding HCl. We then added 10 mL of the pH-adjusted supernatant to 10 mL of fresh ST10^–1^ liquid culture medium, and 10 mL of fresh ST10^–1^ liquid medium to the control treatment. After inoculating BP-3 into this medium, we incubated the cultures as described above. We also performed supernatant amendment experiments on ST10^-1^ agar plates as described previously, with the addition of the bacterial culture supernatant to the medium before it solidified. Additionally, we grew BP-3 overnight in ST10^-1^ liquid culture medium, enriched the culture to an OD_600_ of 1.0, and inoculated the culture onto the centers of ST10^–1^ agar plates (pH 5.0 or pH 7.0) containing 50 mM phosphate, HEPES or MOPS pH-buffers, with bromocresol violet as a pH indicator. The control treatment contained no buffer with the pH of the medium being 7.0. We added both buffer and pH indicator to the medium prior to autoclaving. Finally, we incubated the plates for two days at 30 °C.

### Analysis of organic acids

We cultivated BP-3 in ST10^–1^ medium for three days and collected the resulting supernatant for analysis. We then identified and quantified organic acidic substances in the supernatant by high-performance liquid chromatography coupled with quadrupole-time-of-flight mass spectrometry (HPLC-ESI-qTOF-MS) [42, 43]. To verify the ability of BP-3 to produce pyruvic acid, we cultivated in ST10^-1^ medium with or without 5 mM D-glucose for 2 days, and recorded the OD_600_ and pH of the cultures every six hours. We quantified the concentrations of pyruvic acid in the supernatant of BP-3 by HPLC with details presented in the Supporting Information.

To investigate the role of pyruvic acid in acidifying the ST10^–1^ culture medium, we added 0.6 mM pyruvic acid (equivalent to the amount produced by BP-3 in ST10^-1^ medium) to the medium including glucose and measured the pH. To evaluate the ability of DT-2 to consume pyruvic acid and increase the pH of the culture medium, we added pyruvic acid to the ST10^–1^ medium including glucose to lower the pH to 5.5, and then separately inoculated the DT-2 and BP-3 strains into the medium for cultivation. We measured changes to the pH every 6 h and included a control group with no inoculation.

### Glucose and pyruvate utilization by different strains

To investigate the utilization of glucose and pyruvate by DT-2 and BP-3, we inoculated these strains alone or in co-culture into ST10^–1^ culture medium (without glucose) supplemented with 5.0 mM glucose or 10 mM pyruvate and included control cultures without inoculation. The pyruvate stock solution was prepared prior to utilization by modulating the pH of the 1.0 M pyruvic acid to 7.0 using NaOH. Subsequently, filtration through a 0.22 μM filter was performed, and the resultant solution was introduced into the ST10^-1^ culture medium at pH 7.0 to attain the intended concentrations. Unless specified otherwise, we maintained a consistent procedure for pyruvate addition in other experiments. During the incubation of 48 h, we measured the concentrations of glucose and pyruvate every 6 h using a D-glucose content assay kit (boxbio) and HPLC. Similarly, to assess the effect of glucose and pyruvate on the growth of DT-2, BP-3, CT-33, BT-39, and PN-5, we separately inoculated these strains in ST10^-1^ culture medium (without glucose) supplemented with different concentrations of glucose or pyruvate (0, 0.5, 1, 2, 5, or 10 mM) and recorded the OD_600_ and pH of the cultures after two days.

To evaluate the potential toxicity of sodium pyruvate to bacteria, we investigated its impact on the growth of DT-2, BP-3, CT-33, BT-39, and PN-5. We separately inoculated these strains in ST10^–1^ culture medium (without glucose) supplemented with different concentrations of sodium pyruvate (0, 1, 5, or 10 mM). After two days of incubation, we recorded the OD_600_ of the cultures.

### Multiple sequence alignments and homology structure of dihydrolipoamide acetyltransferase

We performed multiple alignments and generated a homology structure of dihydrolipoamide acetyltransferase (E2) for BP-3, DT-2, and *E. coli* K12 (which contains three lipoyl domains in the E2 protein) using Clustal Omega and AlphaFold2 [44], respectively. As E2 is the core subunit of the pyruvate dehydrogenase multienzyme (PDH) complex, these analyses provide insight into the conservation and structure of this important component.

### Measurement of pyruvate dehydrogenase (PDH) activity

We first incubated DT-2, BP-3, and *E coli*. W3110 into ST10^–1^ culture medium (without glucose) supplemented with 10 mM pyruvate at 30 °C for 12 h. We then quantified the pyruvate dehydrogenase activity of the cells using the PDH activity assay kit (Boxbio), which utilizes a colorimetric method. After centrifuging the growth media at 10,000 g for two minutes, we washed the cell pellets twice with PBS buffer (pH 7.2) and suspended the cells in the solution provided in the assay kit. We then sonicated the cells and collected the supernatant by centrifugation at 10,000 × *g* for 10 min for the PDH activity quantification.

### Transformation of arsenic species

To investigate the transformation of arsenic species by DT-2 and BP-3, we diluted overnight cultures to an OD_600_ of 0.01 in 100 mL fresh ST10^–1^ medium containing either 2 μM MAs(V) or 2 μM MAs(III) and included a control group without inoculation. After 48 h of growth, we centrifuged the growth media at 10,000 × *g* for 2 min and filtering the supernatant through 0.22 μm filters. We then quantified the arsenic species in the filtrates using HPLC coupled with inductively coupled plasma mass spectrometry (HPLC-ICP-MS) [45].

### Assessing the competitiveness of DT-2 and BP-3

To determine whether the MAs(III) generated from MAs(V) reduction by DT-2 had an inhibitory effect on other bacteria, we performed co-culture experiments with BP-3, BT-39, PN-5, or CT-33 on ST10^-1^ agar medium with or without 10.0 μM MAs(V) for two days. We found that all strains varied in their resistance to MAs(III) (Fig. S3).

To assess the competitiveness of strains CT-33, BT-39, and PN-5 against BP-3 and DT-2 for glucose and pyruvate utilization, we performed co-culture experiments. We first grew DT-2, BP-3, BT-39, PN-5, and CT-33 in mono-culture overnight in ST10^–1^ medium and then enriched each mono-culture to an OD_600_ of 1.0 in ST10^–1^ medium without glucose. We then mixed strains DT-2 or BP-3 and CT-33, BT-39, or PN-5 by equal proportions (1%, V/V) in ST10^–1^ aqueous medium (without glucose) with 30 mM glucose or 10 mM pyruvate and incubated the resulting co-cultures for one day. Next, we diluted the mixtures to an OD_600_ of 0.02 in the same medium with glucose or pyruvate and incubated the cultures for an additional day. Finally, we serially transferred the co-cultures every day for six days and measured the density and viability of each strain by colony counting before each transfer.

We also performed tri-culture experiments to assess the advantage of cross-feeding between DT-2 and BP-3 in an environment containing MAs(V). We first grew DT-2, BP-3, BT-39, PN-5, and CT-33 in mono-culture overnight in ST10^–1^ medium and then enriched each mono-culture to an OD_600_ of 1.0 in ST10^-1^ medium without glucose. We then mixed strains DT-2 and BP-3 and further amended these bi-cultures with a third strain (BT-39, PN-5, or CT-33) (1:1:1, V/V) in ST10^-1^ aqueous (1% inoculation) medium with or without 5.0 μM MAs(V) and then incubated the resulting tri-cultures for two days. Next, we diluted the mixtures to an OD_600_ of 0.02 in ST10^-1^ medium with or without 5 μM MAs(V) and incubated the cultures for an additional two days. Finally, we serially transferred the tri-cultures every two days for 12 days and measured the density and viability of each strain by colony counting before each transfer.

### Co-occurrence of *Delftia* sp. and *Bacillus* sp. in soil

To investigate whether the co-occurrence of crossing-feeding strains BP-3 and DT-2 and their close relatives depends on arsenic concentrations in soils, we collected four arsenic-contaminated soils from different regions in China, including Chenzhou (CZ), Hechi (HC), Hengnan (HN), and Tongling (TL), with As concentrations ranging from 42.8 to 249 mg kg^–1^. We assessed their relative abundance in soils amended with varying concentrations of MAs(V) and As(III). Specifically, we placed 100 g aliquots of fresh soil into 500 mL opaque open bottles and added 15 mL of ultra-pure water containing different concentrations of arsenic as MAs(V) or As(III). The final concentrations of arsenic added to the soils were 0, 7, or 15 mg kg^–1^. The control group involved the treatment without any arsenic addition. We then incubated the soils under oxic conditions at 25 °C for 12 days and then extracted total community DNA using the Power Soil DNA Isolation Kit (QIAGEN, Germany). We quantified the abundance of *Delftia* sp., *Bacillus* sp. (DT-2, BP-3, and their close relatives), and total bacterial 16 S rRNA genes by qPCR using specific primer pairs (Table S6) [46], with three replicates for each set. The details for the design of specific primer pairs refer to Supporting Information.

We further collected 103 soil samples from different climatic zones in China that varied in arsenic concentration (5.8 to 158 mg kg^–1^) and quantified the abundances of *Delftia* sp. and *Bacillus* sp. (DT-2, BP-3, and their close relatives) using qPCR with the specific primer pairs (as mentioned above) and bacterial 16 S rRNA sequencing. We also measured the concentrations of soil total arsenic and available inorganic arsenic (iAs) from these soil samples [47].

For bacterial 16 S rRNA sequencing analysis, we used QIIME 1.9.0 [48] to process the sequences (NCBI accession number: PRJNA934706) by quality filtering to remove sequences with barcode mismatches, ambiguous bases, or low-quality reads (Phred quality score <30). After barcode trimming, we performed demultiplexing and utilized the DADA2 pipeline for amplicon sequence variant (ASV) table generation [49, 50]. We employed USEARCH [51] to identify potential chimeras that were excluded for further analysis. We assigned taxonomy using Mothur with the Silva Database [52]. We performed co-occurrence network analysis using the R “igraph” and “psych” packages based on the Spearman’s correlations between the ASVs that were present in at least 10% of the 103 soil samples (*r* > 0.3, *p* < 0.01). To control the false discovery rate, we used the Benjamini-Hochberg method [53].

### Statistical analysis

All data are reported as the mean ± standard error (*n* = 3 ~ 4). We assessed the significance of treatment effects using analysis of variance (ANOVA) and conducted comparisons of treatment means using Tukey’s test (*p* < 0.05). We performed all statistical analyses using IBM SPSS Statistics v. 25.

## Results

### *Delftia* sp. DT-2 and *Bacillus* sp. BP-3 reciprocally promote each other’s growth

**Fig. 1:**
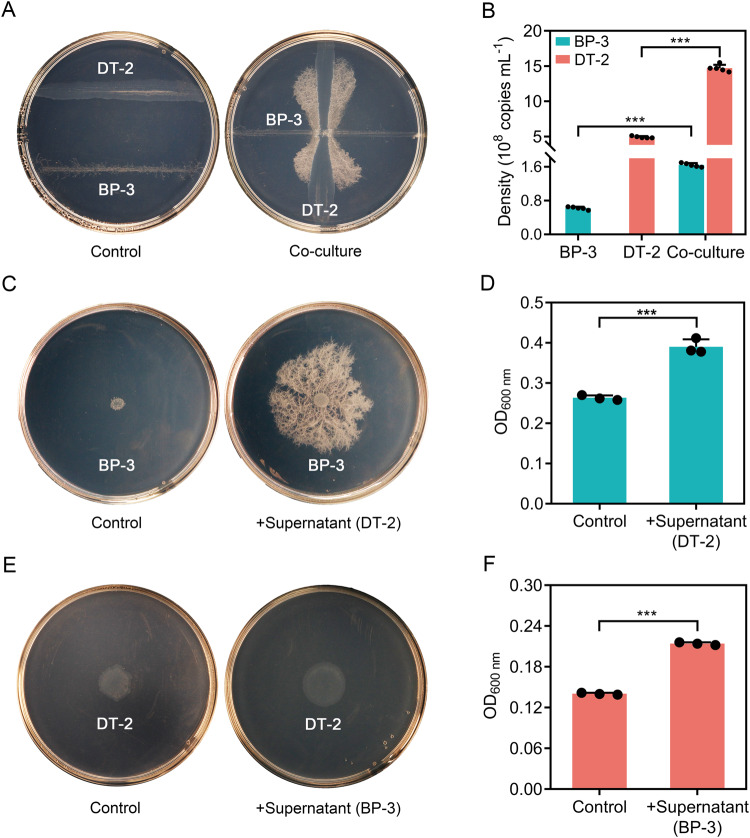
Growth of *Delftia* sp. DT-2 and *Bacillus* sp. BP-3 in mono- or co-culture. The two strains were grown separately or together on ST10^-1^ agar plates (**A**) or in ST10^-1^ liquid medium (**B**). Effects of the culture supernatant of DT-2 on the growth of BP-3 on ST10^–1^ agar plates (**C**) or in ST10^-1^ liquid medium (**D**). Effects of the culture supernatant of BP-3 on the growth of DT-2 on ST10^–1^ agar plates (**E**) or in ST10^–1^ liquid medium (**F**). Ten mL aliquots of the filtered culture supernatant were mixed with 10 mL fresh ST10^–1^ liquid culture medium for inoculation of BP-3 and DT-2. The control group consisted of fresh ST10^–1^ liquid culture medium without supernatant. The strains were grown in the treated culture medium for two days. The data are presented as the mean ± standard error (*n* = 3–4). *** indicates significant differences between treatments at *p* < 0.0001 (Tukey’s test).

During the bacterial isolation process, we found that DT-2 and BP-3 were difficult to separate, as BP-3 colonies tended to only develop when in close proximity to DT-2 colonies (Fig. 1A, Fig. S1, and Video S1). When we streaked DT-2 and BP-3 along separate parallel lines on the same agar plate, both strains exhibited poor growth. However, when streaked the lines such that they intersected perpendicularly, BP-3 grew vigorously and spread from the intersection point along the DT-2 inoculation line, forming a dumbbell shape (Fig. 1A and Video S2). The further from the intersection, the more vigorously BP-3 grew when adjacent to the DT-2 inoculation line. When we cultured DT-2 and BP-3 in mono- or co-culture in liquid medium, both strains reached larger cell densities in co-culture than in mono-culture (Fig. 1B). The abundances of DT-2 and BP-3 in co-culture were 2.0-fold and 1.6-fold higher, respectively, compared to mono-culture. To investigate whether secreted metabolites promoted the observed growth in co-culture, we quantified the effects of culture supernatants on each strain’s growth. DT-2 and BP-3 supernatants significantly stimulated each other’s growth by 48.2% and 52.5%, respectively (Fig. 1C–F). These results demonstrate that secreted metabolites of both strains promote each other’s growth; DT-2 and BP-3 engage in a mutualism where both benefit from each other’s presence.

### *Delftia* sp. DT-2 rescues *Bacillus* sp. BP-3 from self-killing by elevating the pH of the culture medium

**Fig. 2:**
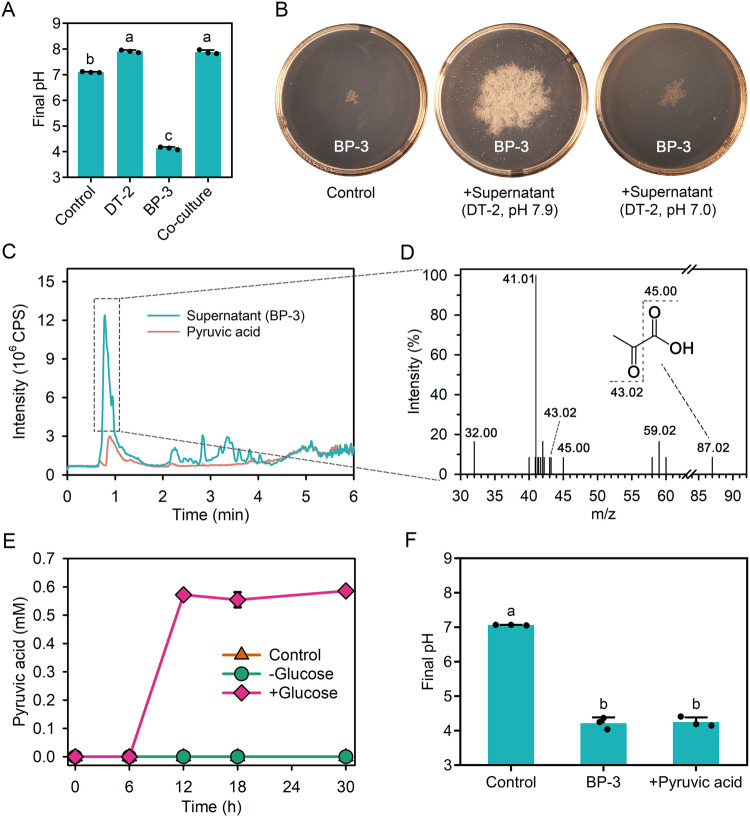
Production of pyruvic acid by *Bacillus* sp. BP-3 and its impact on the acidification of the growth media. **A** Medium pH of DT-2 and BP-3 in mono- and co-culture. A control group consisted of ST10^-1^ culture medium without bacteria. **B** Effect of pH of the DT-2 culture supernatant on the growth of BP-3 on agar plates. BP-3 was grown in the treated culture media for two days. The control group consisted of 10 mL fresh ST10^-1^ liquid medium. **C** LC-MS chromatograms from the supernatant of BP-3. **D** MS spectra of the eluted compound with a retention time from 0.7 to 1.4 min from the supernatant of BP-3. **E** Time course of pyruvic acid concentrations in BP-3 culture medium with or without 5.0 mM glucose. **F** Effect of pyruvic acid (0.6 mM) on the pH of the culture medium. The control group in **E** and **F** used ST10^-1^ medium with 5.0 mM D-glucose but no bacteria. The data are presented as the mean ± standard error (*n* = 3). Error bars in some figures are too small to visualize. Different letters indicate significant differences among treatments at *p* < 0.05 (Tukey’s test).

When grown alone with glucose as the carbon source in liquid medium, almost all of BP-3 died within two days, indicating self-killing (Fig. S4a). The pH of the mono-culture of BP-3 dropped from 7.0 to 4.1 (Fig. 2A), which might explain the self-inhibition of BP-3 growth due to excessive acidification. In comparison, the pH of the mono-culture of DT-2 and the co-culture of DT-2 and BP-3 remained stable around 7.9 by the end of the cultivation (Fig. 2A), demonstrating that DT-2 prevents acidification of the co-culture medium and enables the survival of BP-3. Indeed, the culture supernatant (pH 7.9) from DT-2 significantly promoted the growth of BP-3, whereas the magnitude of the promotion decreased when the pH of the supernatant was adjusted to 7.0 (Fig. 2B and Fig. S5a, b). We further investigated the effect of pH on BP-3 growth by examining its growth on agar plates with different pH buffer systems (Fig. S5c). BP-3 exhibited poor growth in unbuffered medium and medium with buffer systems at pH 5.0, but grew vigorously in various buffer systems at pH 7.0, similar to the growth and pH when co-cultured with DT-2 (Fig. S5c). These results indicate that BP-3 growth becomes self-limited at low pH, while DT-2 counters the acidification of the growth medium, allowing for the prolonged growth of BP-3.

### *Bacillus* sp. BP-3 produces pyruvic acid

To determine the identify of the acid responsible for BP-3’s self-killing, we used HPLC-ESI-qTOF-MS to analyse the metabolites in the mono-culture medium of BP-3. We identified a large peak with a retention time matching that of the pyruvic acid (Fig. 2C). The *m/z* of this peak was 87.02, which matches that of pyruvic acid. The tandem MS spectrum showed the characteristic fragments at *m/z* 43.02 and 45.00, corresponding to CH_3_CO- (*m/z* 43.02) and COOH- (*m/z* 45.00), respectively, of pyruvic acid (CH_3_COCOOH) (Fig. 2D). The *m/z* 41.01 fragment resulted from the loss of two hydrogens from CH_3_CO- (m/z 43.02). These data confirm that the large peak observed in the culture medium of BP-3 was indeed pyruvic acid.

We then quantified the accumulation of pyruvic acid in mono-culture. When we grew BP-3 with 5.0 mM glucose as the carbon source, the concentration of pyruvic acid increased rapidly to 0.6 mM during the first 12 h of incubation (Fig. 2E). This increase was concomitant with the sharp decrease in pH from 7.0 to 4.2 (Fig. 2F). No further changes in the pyruvic acid concentration or pH were observed after 12 h of incubation. In the absence of glucose, we did not detect the production of pyruvic acid (Fig. 2E). Additionally, when we added 0.6 mM pyruvic acid to the base medium (without cells), the pH decreased from 7.0 to 4.2, which was similar to the pH of the mono-culture medium of BP-3 (Fig. 2F). These results confirm that the primary acid produced by BP-3 that leads to acidification of the medium and self-killing is pyruvic acid, which is formed during glucose metabolism.

### Glucose metabolism mediates cross-feeding of *Delftia* sp. DT-2 and *Bacillus* sp. BP-3 via pyruvic acid cross-feeding and pH balancing

**Fig. 3:**
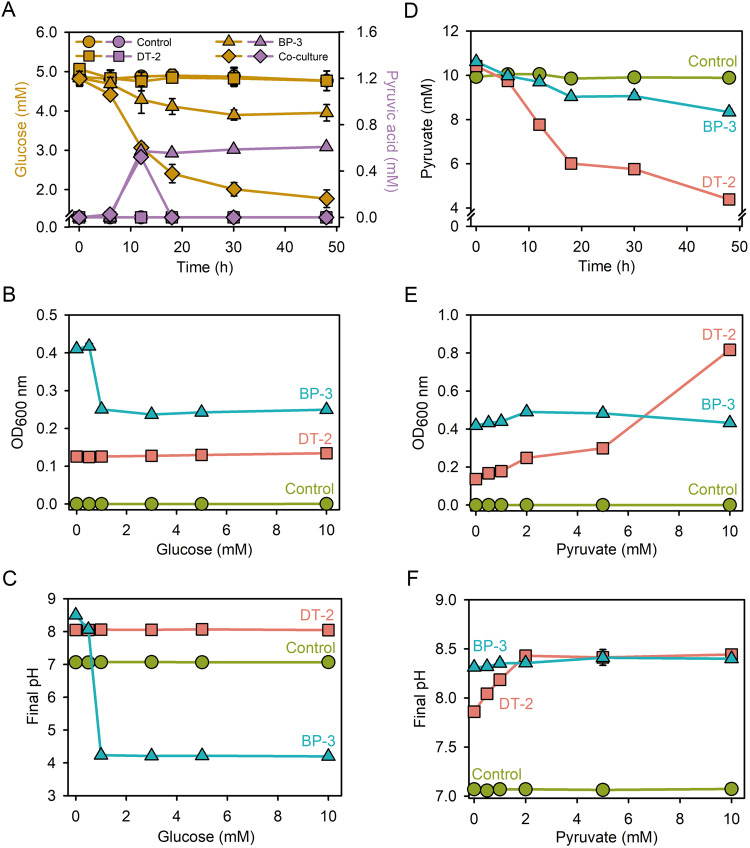
Utilization of glucose and pyruvate by *Delftia* sp. DT-2 and *Bacillus* sp. BP-3. Utilization of glucose (**A**), growth of DT-2 and BP-3 (**B**), and effect on the pH of culture medium (**C**). Utilization of pyruvate (**D**), growth of DT-2 and BP-3 (**E**), and effect on the pH of the culture medium (**F**). The control group did not contain bacteria. Except for the co-culture indicated in panel A, the others were all mono-culture. The data are presented as the mean ± standard error (*n* = 3). Error bars in some figures are too small to visualize.

To investigate whether DT-2 and BP-3 engage in a pyruvic acid cross-feeding and pH balancing interaction, we assessed their glucose utilization traits by inoculating both strains into glucose-amended mono-cultures and co-culture. As expected, DT-2 was unable to grow with glucose (Fig. 3A), while BP-3 consumed glucose from 5.0 mM to 3.9 mM during the first 30 h of incubation, but stopped consuming glucose thereafter, presumably due to pyruvic acid accumulation in mono-culture (Fig. 3A). However, in co-culture, the glucose consumption level of the two strains was significantly higher than that in mono-culture, with glucose concentration decreasing from 5.0 mM to 1.7 mM during the 48 h of incubation (Fig. 3A). Pyruvic acid was detected in the culture medium after 12 h but disappeared soon thereafter, likely due to utilization by DT-2. Pyruvic acid no longer accumulated in the culture medium after 18 h, as DT-2 has a strong pyruvic acid utilization ability.

We also assessed the effects of the initial glucose concentration on the growth of the two strains (Fig. 3B). Glucose at 1.0 mM significantly inhibited growth of BP-3, concomitant with a decrease in pH from 7.0 to 4.2 (Fig. 3C and Fig. S6). Increasing the glucose concentration above 1.0 mM did not increase the growth inhibition of BP-3. In contrast, DT-2 did not use glucose at any of the tested concentrations (Fig. 3A–C).

In addition to glucose, we also investigated the pyruvate utilization traits of the two strains. Both strains were able to consume pyruvate, but DT-2 had a higher consumption rate (Fig. 3D). After 48 h of incubation in mono-culture containing 10.0 mM pyruvate, DT-2 reduced the concentration to 4.4 mM, while strain BP-3 only decreased the concentration to 8.3 mM, indicating that BP-3 is less effective at pyruvate utilization than DT-2 (Fig. 3D). Increasing the initial pyruvate concentration had a stronger effect on the growth of DT-2 than on that of BP-3 (Fig. 3E and Fig. S6). As we increased the initial pyruvate concentration, the OD_600_ of the DT-2 culture gradually increased from 0.14 to 0.82, and the culture pH increased from 7.8 to 8.4 (Fig. 3E, F). This suggests that DT-2 actually consumes pyruvic acid. Conversely, increasing the initial pyruvate had only a weak effect on the growth of BP-3 (Fig. 3E).

To investigate whether the inhibitory effect on the growth of BP-3 (Fig. 3B) was due to the accumulation of pyruvate, we inoculated the strain into sodium pyruvate-amended cultures and varied the concentration of sodium pyruvate. Increasing the concentrations of sodium pyruvate up to 10 mM promoted the growth of BP-3 (Fig. S7), indicating that the self-killing of BP-3 was due to the acidification of the growth environment rather than pyruvate toxicity. Next, we examined whether strain DT-2 could reverse the acidification of the medium caused by pyruvic acid. We cultivated DT-2 in ST10^-1^ medium acidified to 5.5 by exogenously adding pyruvic acid and monitored the pH of the medium. The metabolic growth of DT-2 led to an elevation in the medium’s pH to 7.9 (Fig. S8), affirming its capability to counteract the medium’s acidification attributed to pyruvic acid. The pH elevation is a result of pyruvic acid consumption. Additionally, the ammonia generated from the metabolism of amino acids and peptides might play a role in this pH increase [54, 55].

To investigate why DT-2 and BP-3 differ in their utilization of glucose and pyruvic acid, we analyzed their draft genomes and quantified their pyruvate dehydrogenase activities. We found that hexokinase and phosphotransferase system, two essential enzymes for glucose utilization, are missing from the genome of DT-2. Moreover, the pyruvate dehydrogenase activity of BP-3 was likely much lower than that of DT-2 and *E. coli* W3110 (Fig. S9). We also analyzed the phylogenetic tree based on homolog sequences of dihydrolipoamide acetyltransferase (E2), a subunit of the pyruvate dehydrogenase complex. The phylogeny and protein alignment confirmed the identity of E2 of BP-3 as within known dihydrolipoamide acetyltransferase (Figs. S10 and S11). Further analysis of the amino acid sequence and structure of the E2 protein using AlphaFold2 revealed that the N-terminus of E2 in BP-3 contained only one lipoyl domain, whereas E2 proteins of DT-2 and *E. coli* K12 contain two and three lipoyl domains, respectively (Figs. S11 and S12). This suggests that the low activity of pyruvate dehydrogenase in BP-3 (Fig. S9) is likely due to having only a single lipoyl domain.

Our findings indicate that the differences in the metabolism of glucose and pyruvic acid are the basis of the mutualistic interaction between DT-2 and BP-3 (Figs. 1–3). When grown in mono-culture, BP-3 uses glucose and produces a large amount of pyruvic acid, which accumulates and acidifies the growth environment leading to self-killing (Fig. S4a). However, in co-culture, DT-2 readily utilizes the pyruvic acid produced by BP-3 as a carbon source, promoting its own growth and preventing acidification of the medium, thus rescuing BP-3 from self-killing. This cross-feeding mechanism is not unique to DT-2 and BP-3, as the randomly picked soil strains CT-33 and BT-39 also produce large amounts of pyruvic acid (Figs. S13 and S14b) that lead to self-killing (Fig. S4b, c). Additionally, these two strains also able to promote each other’s growth with DT-2, indicating the same cross-feeding mechanism (as illustrated in Fig. S4b, c).

### *Delftia* sp. DT-2 and *Bacillus* sp. BP-3 participate in a second mutualistic interaction involving arsenic transformation

In preliminary experiments, we observed a higher frequency of co-occurrence of BP-3 and DT-2 in the presence of arsenicals. Further investigation of the draft genomes of the two strains revealed an arsenite methyltransferase gene (*BparsM*) encoding an ArsM homolog in BP-3 (Fig. S15a), whose expression is induced by MAs(III) or As(III) (Fig. S15c, d). Heterologous expression of *BparsM* conferred MAs(III) resistance to *E. coli* AW3110, and purified BpArsM was able to methylate MAs(III) (Fig. S16).

**Fig. 4:**
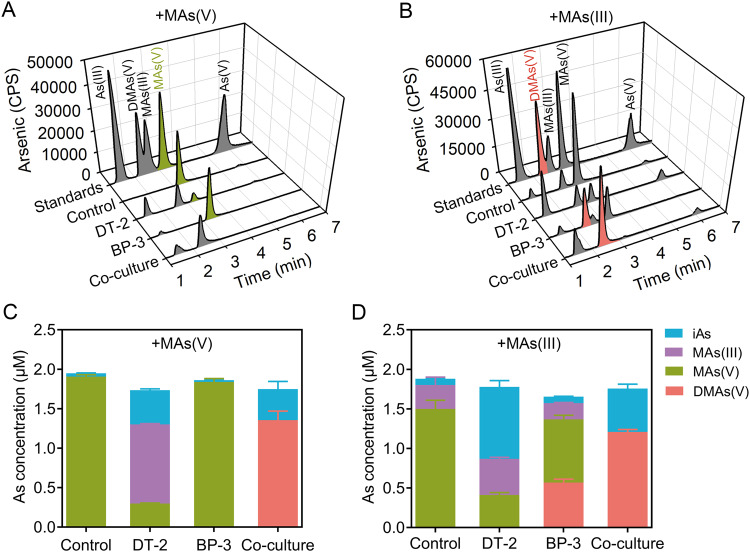
Transformation of arsenic compounds by *Delftia* sp. DT-2 and *Bacillus* sp. BP-3 in mono- or co-culture. Transformation of MAs(V) (**A,** **C**) and MAs(III) (**B,** **D**) by DT-2 and BP-3 in mono- and co-culture. The two strains were grown in ST10^–1^ culture medium containing 2.0 μM MAs(V) or MAs(III) for two days. The control group did not contain bacteria. Different arsenic species were determined by HPLC-ICP-MS. The data are presented as the mean ± standard error (*n* = 3).

We next investigated the transformation of As species in mono- and co-cultures of DT-2 and BP-3 in the presence of either MAs(V) or MAs(III). In mono-culture, DT-2 reduced MAs(V) to MAs(III) and demethylated a small amount of MAs(III) to As(III) (Fig. 4A, C), but did not further methylated MAs(III) to DMAs(V) (Fig. 4b, d). In contrast, mono-cultures of BP-3 could not reduce MAs(V) (Fig. 4A, C), but was able to methylate MAs(III) to DMAs(V) (Fig. 4B, D).

When co-cultured, the two strains established an As speciation transformation interaction. In co-culture of DT-2 and BP-3 with the addition of MAs(V), DT-2 reduced most of the MAs(V) into MAs(III) while BP-3 further methylated it into DMAs(V) (Fig. 4A, C and Fig. S17). BP-3 promoted the reduction of MAs(V) to MAs(III) by DT-2 (Fig. 4A, C). MAs(III) was readily oxidized to MAs(V) under oxic conditions, as shown in the control treatment (Fig. 4B, D). In co-culture of DT-2 and BP-3 with the addition of MAs(III), the activity of DT-2 promoted the methylation of MAs(III) to DMAs(V) by BP-3 when compared with the mono-culture of BP-3 (Fig. 4B, D). One possible reason that DT-2 promotes the methylation of MAs(III) by BP-3 is because DT-2 efficiently reduces the oxidized MAs(V) to MAs(III), which is then used as the substrate for further methylation by BP-3. These results indicate that DT-2 and BP-3 mutually promote each other’s arsenic transformation.

### Mutualistic arsenic transformation interaction between *Delftia* sp. DT-2 and *Bacillus* sp. BP-3 confers a competitive advantage for the strains

We conducted experiments to evaluate the utilization characteristics of glucose and pyruvate by soil bacteria CT-33, BT-39, and PN-5. We inoculated each strain in mono-culture with varying concentrations of glucose or pyruvate and found that increasing concentrations of either glucose or pyruvate significantly promoted the growth of all three strains (Fig. S14). We further investigated the utilization effectiveness in co-cultures of BP-3 with CT-33, BT-39, or PN-5 in the presence of glucose, and co-cultures of DT-2 with CT-33, BT-39, or PN-5 in the presence of pyruvate. In the co-cultures with BP-3 and the three competitors in the presence of glucose, the frequency of BP-3 decreased significantly, while the frequencies of CT-33, BT-39, and PN-5 gradually and significantly increased (*p* < 0.05) (Fig. S18). This suggests that the three competitors had a clear growth advantage over BP-3 and were able to outperform BP-3 in utilizing glucose. In co-cultures of DT-2 with CT-33, BT-39, or PN-5 in the presence of pyruvate, the frequency of DT-2 decreased significantly while the frequency of PN-5 gradually and significantly increased (*p* < 0.05) (Fig. S18). This suggests that PN-5 had an advantage over DT-2 in pyruvate utilization. However, strains CT-33 and BT-39 did not have an advantage over DT-2 in pyruvate utilization.

**Fig. 5:**
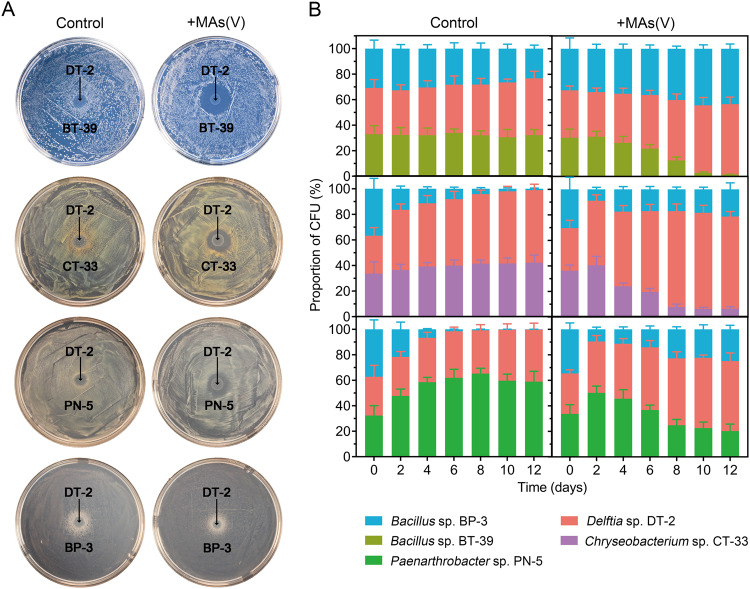
Growth of communities consisting of two or three strains with or without the presence of MAs(V) in the growth media. **A** *Delftia* sp. DT-2 was co-cultured with *Bacillus* sp. BP-3, *Bacillus* sp. BT-39, *Paenarthrobacter* sp. PN-5, or *Chryseobacterium* sp. CT-33 on ST10^–1^ agar plates with or without 10 μM MAs(V). **B** DT-2, BP-3, and a third strain (BT-39, PN-5, or CT-33) were co-cultured in liquid medium with or without 5.0 µM MAs(V). The control groups were treatments without MAs(V). The data are presented as the mean ± standard error (*n* = 3).

The collaboration between DT-2 and BP-3 in reducing MAs(V) to MAs(III) and subsequently methylating it may give them an edge over other microbes because MAs(III) has antimicrobial activity [23]. We evaluated this hypothesis by co-culturing DT-2 with different soil bacteria (BP-3, BT-39, PN-5, or CT-33) on ST10^-1^ agar plates, both with or without MAs(V), for two days. In the medium without MAs(V), BP-3, BT-39, PN-5, and CT-33 grew well in proximity to DT-2 without forming a zone of inhibition (Fig. 5A). However, in the medium containing MAs(V), BT-39, PN-5, and CT-33 displayed distinct zones of inhibition, while BP-3 grew actively next to DT-2 without any inhibition zone. These findings demonstrate that MAs(III) generated from MAs(V) reduction by DT-2 suppressed the growth of BT-39, PN-5, and CT-33, while BP-3 remained unaffected.

We assessed the growth of co-cultures of DT-2, BP-3, and a third strain (BT-39, PN-5, or CT-33) with or without MAs(V). In co-cultures without MAs(V), the frequency of the third strain remained stable (BT-39 and CT-33) or considerably increased (PN-5), whereas the frequency of BP-3 gradually and significantly decreased (*p* < 0.05) in the co-culture with CT-33, PN-5, or BT-39 (Fig. 5B). In co-cultures with MAs(V), the frequencies of both DT-2 and BP-3 significantly increased (*p* < 0.05), while those of BT-39, CT-33, and PN-5 decreased significantly (*p* < 0.05). For example, after 12 days of incubation, the frequencies of DT-2, BP-3, and BT-39 were 45%, 23%, and 32%, respectively, in co-cultures without MAs(V). These frequencies changed significantly (*p* < 0.05) to 55%, 43%, and 2%, respectively, in the presence of MAs(V) (Fig. 5B). These results demonstrate that the interaction between DT-2 and BP-3 in arsenic transformation confers them a competitive advantage over other members of the microbial community.

### Co-occurrence of *Delftia* sp. and *Bacillus* sp. is prevalent in soils

**Fig. 6:**
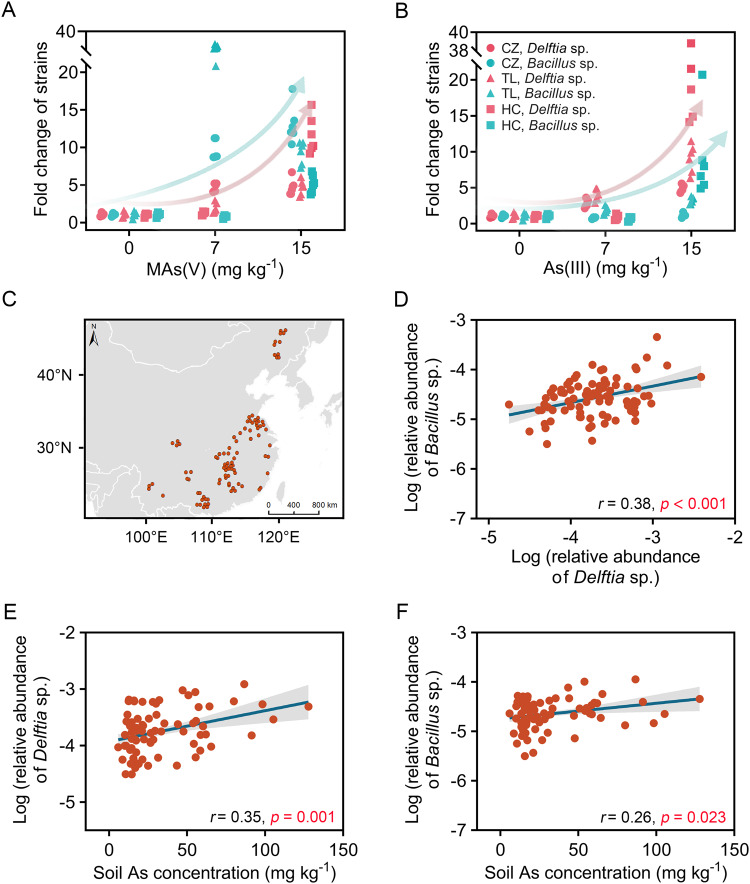
Co-ooccurrence of *Delftia* sp. and *Bacillus* sp. is prevalent in soil environments and is influenced by soil arsenic concentrations. The abundance changes of *Delftia* sp. and *Bacillus* sp. in soils amended with different concentrations of arsenic as MAs(V) (**A**) or As(III) (**B**). The comparative control entails the treatment without the addition of arsenic. **C** Maps of 103 soil samples collected from different climatic zones in China. **D** Correlation between the relative abundances of *Delftia* sp. and *Bacillus* sp, with points showing the co-occurrence in 88 soil samples. Correlations of relative abundances of *Delftia* sp. (**E**) and *Bacillus* sp. (**F**) with soil arsenic concentrations.

We assessed the prevalence of co-occurrence of *Delftia* sp. and *Bacillus* sp. (DT-2, BP-3, and their close relatives, see below) in soils to determine how common the two-tiered mutualism may be in nature. After 12 days of cultivation, we detected *Delftia* sp. and *Bacillus* sp. in three of the four tested soils using specific primers (Fig. 6A, B). The addition of MAs(V) or As(III) to the soils increased the abundances of both bacteria in a dose-dependent manner (Fig. 6A, B). For instance, in the CZ soil, the addition of 15 mg As kg^-1^ as MAs(V) significantly increased the relative abundances of both *Delftia* sp. and *Bacillus* sp. by 4.7- and 13.1-fold, respectively, compared to the control (no added arsenic). (Fig. 6A). Similarly, the addition of 15 mg As kg^-1^ As(III) significantly increased the relative abundance of *Delftia* sp. by 4.8-fold, with less effect on growth of *Bacillus* sp. (Fig. 6B).

We investigated whether *Delftia* sp. and *Bacillus* sp. co-occur in 103 soils collected from different climatic zones in China (Fig. 6C). We detected *Delftia* sp. and *Bacillus* sp. in 96 and 92 soils, respectively, with the two types of bacteria coexisting in 88 soils. The relative abundances of the two types of bacteria were positively and significantly correlated (*r* = 0.38, *p* < 0.001) (Fig. 6D), which is what would be expected were a mutual interdependence occurring between the two types of bacteria. Moreover, we observed significant positive correlations between the relative abundances of *Delftia* sp. or *Bacillus* sp. and soil total arsenic and available inorganic arsenic concentrations (Fig. 6E, F and Fig. S19). We confirmed these correlations by 16 S rRNA gene data, which clearly identified correlations between the abundances of *Delftia* sp. and *Bacillus* sp. and with soil arsenic concentration (Fig. S20).

Genes and interactions are conserved in species that are phylogenetically closely related to DT-2 or BP-3. By using the KEGG pathway and NCBI GenBank database, we observed that several soil bacteria closely related to DT-2 (Fig. S2a), such as *Delftia acidovorans, Delftia lacustris, and Delftia tsuruhatensis*, are predicted to lack hexokinase and phosphotransferase systems but possess a complete pyruvic acid metabolic pathway (pyruvate dehydrogenase, dihydrolipoamide acetyltransferase, and dihydrolipoamide dehydrogenase) (Fig. S21). These bacteria also harbor the glutamate-cysteine ligase gene (*gshA*). This gene plays a vital role in the reduction of MAs(V) to MAs(III) [24], suggesting that these species possess the potential for MAs(V) reduction. Conversely, species related to BP-3 (Fig. S2b), including *Bacillus anthracis*, *Bacillus cereus*, *Bacillus pseudomycoides*, and *Bacillus nitratireducens*, have complete glucose metabolism pathways and can utilize glucose to produce organic acids, including pyruvic acid (Fig. S22). These observations suggest that many *Bacillus* species may have the ability to generate pyruvic acid from glucose, thereby acidifing their growth environment [15, 56–59]. These bacteria also contain the *arsM* gene in their genomes, which enables the methylation of MAs(III). These analyzes suggest that these metabolic potentials are conserved among bacteria closely related to DT-2 or BP-3.

To provide further support for this generality, we analysed 16 S rRNA data from the 103 soils to identify bacteria whose abundances are positively correlated with soil arsenic concentrations (Fig. S20). We identified 1324 paired bacteria and found that their relative abundances were significantly and positively correlated. Remarkably, pairs such as *Gemmata* sp. and *Gemmatimonas* sp., *Methylocystis* sp. and *Gemmatimonas* sp., and *Methylocystis* sp. and *Bacillus* sp. showed significant associations with soil arsenic concentrations (*r* > 0.3, *p* < 0.01). Based on the KEGG pathway database, we found *Methylocystis* sp. and *Gemmata* sp. lack a hexokinase and phosphotransferase system but possess a complete pyruvic acid metabolic pathway [60]. As a result, they cannot utilize glucose as a carbon source but can rely on organic acids such as acetic, pyruvic, malic, and succinic acids [61, 62]. In contrast, *Gemmatimonas* sp. and *Bacillus* sp. have a complete glycolytic metabolic pathway and use glucose to produce a variety of organic acids [63–65]. Additionally, these co-occurring species also exhibit potential interactions related to arsenic transformations based on their reported genomes (NCBI GenBank database). For instance, the genomes of *Gemmata* sp. and *Methylocystis* sp. contain the *gshA* gene [60], while their corresponding pairs *Gemmatimonas* sp. and *Bacillus* sp. harbor the *arsM* gene [63, 64]. These ecological analyses support the idea that the two-tiered cross-feeding mechanism involving carbon metabolism and arsenic transformation interactions is likely widespread in soil ecosystems.

## Discussion

We have isolated two naturally occurring cross-feeding strains, BP-3 and DT-2, which exhibit distinct metabolic traits and mutually support each other’s growth (Fig. 1, Video S1 and S4a). The two strains, along with their closely related counterparts, are widely distributed in soil environments, as demonstrated by their co-occurrence in 88 out of the 103 soils tested (Fig. 6D). These two types of strains belong to species known as plant growth-promoting rhizobacteria (PGPR) and biocontrol bacteria [66], and they are frequently encountered and exhibit co-occurrence within microbial communities present in soil ecosystems with relatively high abundance [67–73]. However, these two types of strains differ in their carbon source utilization capabilities. For example, DT-2 is unable to utilize glucose, which is likely due to the absence of hexokinase and phosphotransferase systems, while BP-3 exhibits limited pyruvic acid utilization, which is possibly due to its low pyruvate dehydrogenase activity (Fig. 3D, E and Fig. S9).The N-terminus of E2 in BP-3 contains only a single lipoyl domain (Figs. S11 and S12). The lipoyl domains is important for the activity of pyruvate dehydrogenase, and an increased number of lipoyl domains from one to three substantially increases enzymatic activity and increases carbon conversion efficiency [74–76]. Therefore, the low activity of pyruvate dehydrogenase in BP-3 (Fig. S9) is likely attributable to having only a single lipoyl domain.

**Fig. 7:**
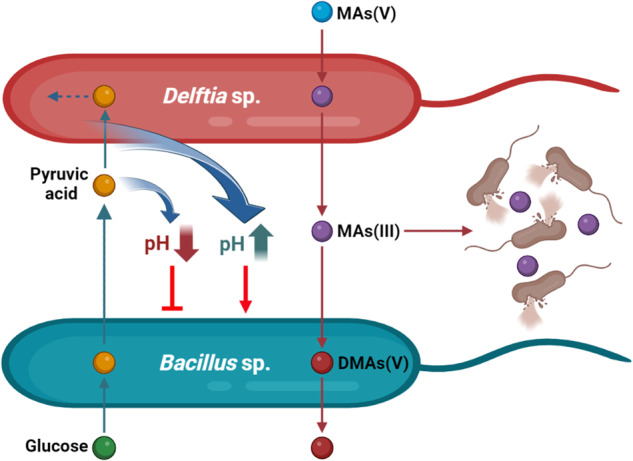
Illustrative model of the cross-feeding interactions between *Bacillus* sp. and *Delftia* sp. in carbon metabolism and arsenic transformation. The two-tiered mutualsm enables the cross-feeders to gain a competitive advantage in the environment.

Pyruvic acid is a crucial intermediate in bacterial sugar metabolism and the interconversion of organic intermediates in cells. However, the inability of BP-3, CT-33, and BT-39 to efficiently utilize pyruvic acid results in its accumulation in the growth medium, leading to acidification and self-killing of these strains (Fig. 3B, C, Fig. S4 and S13). *Bacillus* sp. B12, *Bacillus* sp. B38, and *Bacillus* sp. B36 also acidify the ST10^-1^ medium during the utilization of glucose, lowering the pH of their growth environment (Fig. S13). This phenomenon has been observed in other bacteria that secrete organic acids from glucose metabolism, such as lactic, acetic, succinic, and pyruvic acid, causing rapid extinction of the entire population due to the lowered pH of the environment [14, 15, 56, 77–79]. In contrast, DT-2, with a defective hexokinase and phosphotransferase system, efficiently utilizes pyruvic acid as a carbon source (Fig. 3D–F), removing the acid from the growth medium and raising environmental pH to prevent the self-killing of BP-3, CT-33, and BT-39 (Fig. 2A, Figs. S4, S8 and S13). Many bacteria, such as *Rhodopseudomonas* sp., *Acinetobacter* sp., and *Delftia* sp. are often defective in hexokinase and phosphotransferase system and consequently cannot utilize glucose [80–83]. They are able to utilize a variety of organic acids including pyruvic acid. An artificial combination of *E. coli* and *Rhodopseudomonas palustris* was previously found to cross-feed organic acids and ammonium, maintaining an obligate symbiotic relationship between the two strains [14]. In contrast, *Delftia* sp. and *Bacillus* sp. are naturally-occurring cross-feeding strains in soil, and their relative abundances are positively correlated (Fig. 6C, D and Fig. S20). Our results demonstrate that cross-feeding coupled with pH homeostasis mitigates bacterial defects in carbon source utilization and promotes each other’s growth (i.e., the Tier I mutualism, Fig. 7). The Tier I mutualism allows the two types of strains to overcome the metabolic defects and prevalently coexist in the community.

DT2 and BP-3 engage not only in a carbon metabolism-based mutualism but also in a second arsenic transformation-based mutualism. DT-2 reduces MAs(V) to highly toxic MAs(III) that is then methylated to DMAs(V) by BP-3 when these two strains co-occur, thereby enhancing the two steps of arsenic biotransformation (Fig. 4). This is a mutualistic process of sequential antimicrobial production and detoxification. The production of antimicrobial MAs(III) by this mutualism effectively eliminates or impairs competing bacteria. To maintain the cross-feeding relationship with DT-2, BP-3 must possess the ability to tolerate or detoxify MAs(III). BP-3 achieves this by employing ArsM, which methylate MAs(III) into relatively non-toxic DMAs(V) (Fig. S16). The capacity to detoxify MAs(III) ensures the survival of the cross-feeding strains and enables them to utilize arsenic as a weapon in microbial warfare, thereby gaining a competitive growth advantage (Figs. 5 and 6).

Microbes have been exposed to arsenic since the inception of life and have evolved the ability to both produce arsenical antimicrobials and detoxify them [22, 23, 25, 30]. The production of MAs(III) as a bioweapon allows producers to eliminate competitors and exploit limited environmental resources [23, 24, 26, 28]. Following the Great Oxidation Event, MAs(III) oxidized to MAs(V) in the presence of air [84]. Many bacteria have evolved the capability to reduce relatively nontoxic MAs(V) to highly toxic MAs(III), which creates antimicrobial stress for susceptible bacteria [23, 85, 86]. *ArsM* genes, widely present in microbial genomes, enable the detoxification of MAs(III) through methylation [23, 25, 30, 87]. In soil environments, MAs(V) is an ubiquitous arsenic species that can arise from the methylation of As(III) or applications of As-containing herbicides and defoliants [27, 28, 39], providing a substate for soil microbes to generate MAs(III). In the present study, DT-2 and BP-3 are at a competitive disadvantage due to defects in energy metabolism and carbon utilization (Fig. 5B). However, the presence of arsenic increases their survival advantages, as evidenced by their increased abundance with increasing soil arsenic (Figs. 5B and 6E, F). These results indicate that the two cross-feeders have evolved a bioweapon through production of MAs(III), which provides them an evolutionary advantage that compensates for their energy metabolism defects (i.e., the Tier II mutualism, Fig. 7). The Tier II mutualism enables the two types of cross-feeding strains to outcompete other microbes in arsenic-contaminated environments.

In the Tier II mutualism, the production of highly toxic arsenic and detoxification processes in DT-2 and BP-3 can be seen as a form of “regulation” against cheaters or competing organisms [88, 89]. This regulation prevents the utilization of glucose and pyruvate by other bacteria, which is beneficial in the Tier I mutualism. In our study, three competing strains, CT-33, BT-39, and PN-5, demonstrated superior utilization of glucose compared to BP-3, while PN-5 outperformed DT-2 in utilizing pyruvate (Figs. S14 and S18). However, when arsenic is present, the cross-feeder is capable of using arsenic to suppress the growth of these competing resource-utilizing organisms (Fig. 5). While arsenic resistance genes are prevalent on a global scale, it is important to note that there are numerous soils where such genes are absent [90]. In the environment, there exists a considerable number of bacteria sensitive to MAs(III), as observed in strains like BT-39, CT-33, and PN-5 studied here, as well as previously reported cases involving bacteria such as *B. subtilis*, *E. coli*, and *Staphyloccocus aureus* [91, 92]. The interplay between antibiotic-producing and tolerant bacteria in the environment could confer a competitive edge to the widespread MAs(III)-susceptible strains. It is important to note that there are some differences between the phenomenon observed in this study and previous microbial cheater control mechanisms. Previous reports on cheaters mainly involve mutants within a single species population that cease to perform certain functions that are costly to the individual but beneficial to the community, thereby gaining a fitness advantage at the expense of the conspecifics [89, 93, 94]. In contrast, the behavior of regulation against cheaters observed in this study occurs between different species, involving a dynamic process with multiple species interactions.

The behavior of these cross-feeders aligns with key predictions of the “Black Queen Hypothesis” (BQH) [95, 96]. The BQH provides a simplified theory of dependent evolution in microbial communities, explaining how certain bacteria, lacking certain adaptive genes, exploit common resources leaked by other bacteria. It elucidates how natural selection can promote the loss of metabolic functions and the emergence of mutualistic interactions. Black Queen (BQ) traits are critical for the survival of the microbial community, but only some members bear the cost of these traits, while other members lose this expensive trait, saving energy costs [97]. The BQH may be prevalent in the environment, supported by evidence from various microbiomes, and may be a powerful lens for examining the ecology of microbiomes [95–98]. Once a single mutualistic interaction emerges, it imposes selective pressure on the interacting genotypes to maintain prolonged spatial proximity with each other. This prolonged spatial proximity then sets the stage for further gene loss and the emergence of multi-tiered mutualisms. In our case, the lack of hexokinase and phosphotransferase systems in DT-2 and the poor catalytic activity of pyruvate dehydrogenase in BP-3 likely arose from reductive evolutionary processes, as the main pathways are otherwise complete. This scenario may have then set the stage for prolonged spatial proximity of these two strains and the further loss of their arsenic transformation capabilities, ultimately resulting in the emergence of the two-tiered mutualism.

In conclusion, our study reveals a novel two-tier mutualism between *Delftia* sp. and *Bacillus* sp., two types of soil bacterial strains. These strains cross-feed by compensating for each other’s metabolic defects in glucose and pyruvic acid utilization, and they gain a competitive advantage in the microbial community by producing and detoxifying an arsenical antimicrobial. These mutualistic interactions enhance their survivability in arsenic-containing environments. Considering that arsenic is ubiquitous in the environment, cross-feeding based on carbon metabolism and the use of arsenic as a weapon in microbial warfare are likely common microbial interactions that shape microbial community structure and maintain the stability and diversity.

### Supplementary information

Supplemental Material

Supplemental Video S1

Supplemental Video S1

## Data Availability

All data reported in this paper will be shared by the lead contact upon request.
