# Supplementary material for: Two-tiered mutualism improves survival and competitiveness of cross-feeding soil bacteria
Source: ISME J. 2023 Sep 22;17(11):2090–102. doi: 10.1038/s41396-023-01519-5 (PMC10579247; doi:10.1038/s41396-023-01519-5)
Supplement: Supplementary file 1 — Supplemental Material [file 41396_2023_1519_MOESM1_ESM.docx]

**Supporting Information**

**A two-tiered mutualism improves the survival and competitiveness of cross-feeding bacteria in soil**

Zhan-Biao Ge^1,2^, Zhi-Qiang Zhai^1,2^, Wan-Ying Xie^1^, Jun Dai^1^, Ke Huang^1^, David R. Johnson^3,4^, Fang-Jie Zhao^1^, and Peng Wang^1,2,*^

*^1^ Jiangsu Collaborative Innovation Center for Solid Organic Waste Resource Utilization, College of Resources and Environmental Sciences, Nanjing Agricultural University, Nanjing 210095, China*

*^2^ Centre for Agriculture and Health, Academy for Advanced Interdisciplinary Studies, Nanjing Agricultural University, Nanjing 210095, China*

*^3^* *Department of Environmental Microbiology, Swiss Federal Institute of Aquatic Science and Technology (Eawag), 8600 Dübendorf, Switzerland*

*^4^ Institute of Ecology and Evolution, University of Bern, 3012 Bern, Switzerland*

**35 pages in total**

**2 videos**

**6 tables**

**22 figures**

**Materials and Methods**

**qPCR to assess the growth of DT-2 and BP-3.** Real-time quantitative PCR (RT-qPCR) was used to assess the growth of *Delftia* sp. DT-2 and *Bacillus* sp. BP-3. Total DNA were extracted from the cultures using a bacterial DNA kit (OMEGA) and used for draft genome sequencing in biozeron Co., Ltd. (Shanghai, China). The copy numbers of the *ArsI* and *gerM* genes were used as proxies for the growth of DT-2 and BP-3, respectively. Copy numbers were measured using SYBR Green Master Mix (Vazyme) on a CFX96 Thermocycler (Bio-Rad, USA). The DNA concentration and purity were determined using a NanoDrop 2000C spectrophotometer (Thermo Scientific, USA). The RT-qPCR primers used are listed in Table S3. The 20 μL RT-qPCR reaction system contained 10 µL 2×AceQ qPCR SYBR green Master Mix, 0.4 µL of each primer (200 nM), and 2 µL DNA template (10 ng). Each set contains six replicates. The PCR parameters were as follows: 5 min at 95 °C; 40 cycles of 15 s at 95 °C; 30 s at 55 °C; and 30 s at 72 °C. The specificity of amplification was confirmed by examining the post-PCR melting curves. The copy number of each gene in each sample was determined by using the standard curves, as described previously [1, 2].

To construct the standard plasmids, genomic DNA from both strains was obtained and used as the template for PCR. PCR was performed using specific primer pairs listed in Table S3 to generate gene fragments of *arsI* and *gerM*. The gene fragments were then ligated into the pEASY®-T3 vector by TA cloning. Plasmids pEASY-*arsI* and pEASY-*gerM* were obtained using a plasmid Mini kit (OMEGA). The DNA concentrations of the plasmids were determined using a NanoDrop 2000 spectrophotometer. The copy numbers of the plasmids were calculated [1, 2]. The plasmids were serially diluted by 10-fold and used as templates to generate standard curves. The PCR amplification efficiency was determined using the standard curves. All amplifications exhibited an efficiency within the range of 90% to 110%.

**Analysis and quantitative of organic acids.** We identified and quantified organic acidic substances in the supernatant of BP-3 by high-performance liquid chromatography coupled with quadrupole-time-of-flight mass spectrometry (HPLC-ESI-qTOF-MS) [3, 4]. To confirm the ability of BP-3, CT-33, BT-39, and PN-5 to produce pyruvic acid, we grew these strains in ST10^-1^ medium with or without 5.0 mM D-glucose for two days and measured the pH of the cultures every six hours. A control group was also included, using ST10^-1^ medium with 5.0 mM D-glucose but no bacterial inoculation. We quantified the concentrations of pyruvic acid in the culture supernatants at a 6-h interval using HPLC [5]. We performed HPLC quantitative analysis on an Agilent 1100 system with a Waters Atlantis C18 column (5 μm, 150 mm × 4.6 mm) and a UV diode array detector set at 220 nm. We separated pyruvic acid using a mobile phase consisting of 25 mM KH_2_PO_4_, adjusted to pH 2.5 with orthophosphoric acid and methanol (95:5, V/V). We set the flow rate and injection volume to 1.0 mL min^-1^ and 50 μL, respectively.

**Cloning, expression, and purification of BpArsM.** Based on the draft genome of *Bacillus* sp. BP-3, we identified a putative type methyltransferase gene (*BparsM*), which encodes an ArsM homolog (Figure S15). To analyze the nucleotide and deduced amino acid sequence of BparsM, we conducted Nucleotide BLASN and BLASTX seraches. Multiple alignments of ArsM homolog sequences were performed using Clustal Omega (http://www.ebi.ac.uk/Tools/msa/clustalo/). To obtain the *BparsM* gene, we amplified it from the genomic DNA of BP-3 using the primer pair *BparsM*-F and *BparsM*-R (Table S5). The PCR products were then digested with *Nde*I and *Xho*I and inserted into the corresponding sites of the pET29a plasmid, which is under the control of the T7 promoter. Subsequently, we transformed the resulting pET29a-*BparsM* plasmid into *E. coli* BL21 or AW3110 for further analysis.

Protein purification was conducted as follows: *E. coli* BL21 cells carrying the pET29a-*BparsM* plasmid were inoculated into LB culture medium supplemented with 50 μg mL^-1^ kanamycin. After reaching an optical density at OD_600_ of 0.5, the expression of BpArsM was induced by adding 0.3 mM isopropyl β-D-1-thiogalactopyranoside (IPTG). The cultures were further grown at 16 °C for 16 h. Cells were harvested by centrifugation at 10,000 g, washed twice with 20 mL of buffer solution A (20 mM Na_3_PO_4_, 0.5 M NaCl, pH 7.4) and resuspended in 25 mL buffer A. The cells were lysed using sonication, and the cell debris was removed by centrifugation at 10,000 g for 40 min. The supernatant was then filtered through a 0.45 μm MCE syringe filter (ASD, China) and loaded onto a pre-equilibrated Ni-NTA column (Sangon Biotech Shanghai) containing 50 mL buffer A with 10 mM imidazole. The column was washed with 50 mL of buffer A containing 20 mM imidazole and the protein was eluted with 20 mL of buffer A containing 200 mM imidazole. The purified protein was analyzed using sodium dodecyl sulfate polyacrylamide gel electrophoresis (SDS-PAGE), and the protein concentrations was determined using a Nanodrop-2000C spectrophotometer. The purified BpArsM protein was stored at -80 °C until further use.

**RT-qPCR for** **the expression of *BparsM* gene.** To investigate whether the expression of *BparsM* gene is affected by arsenic concentrations, we grew BP-3 in ST10^-1^ culture medium at 30 °C until mid-exponential phase in the presence of varying concentrations of arsenic, either as MAs(III) (0, 1, 3, or 5 μM) or As(III) (0, 1, 3, or 10 μM). Total RNA was extracted using a bacterial RNA isolation kit (OMEGA), and RNA concentrations was measured using a NanoDrop 2000 spectrophotometer. Subsequently, we synthesized cDNA using the HiScript Reverse Transcriptase kit (Vazyme) and used it as the template for RT-qPCR analyses. The primer sequences are listed in Table S4. The relative expression levels were quantified using the 2^-ΔΔCT^ method [6].

**Arsenic methylation activity of BpArsM.** To assess the methylation activity of BpArsM, we first purified the protein following the previously described protocol [7]. Subsequently, we incubated the purified BpArsM protein with either As(III) or MAs(III) substrates in PBS buffer (20 mM KH_2_PO_4_, 20 mM Na_2_HPO_4_, 0.15 M NaCl, pH 7.4) at 30 °C, supplemented with 8.0 mM GSH and 1.0 mM SAM. Following the incubation period, we terminated the reactions by adding 6% (v/v) H_2_O_2_ and subjecting them to heat treatment at 80 °C for 5 min to oxidize all arsenic species. The resulting solution samples were then filtered through 0.22 μm MCE syringe filters, and the filteres were analyzed using HPLC-ICP-MS.

**MAs(III) tolerance assay.** To investigate the MAs(III) resistance conferred by *BparsM*, we grew *E. coli* AW3110 strains carrying either plasmid pET29a-*BparsM* or plasmid pET29a in LB culture medium supplemented with 50 μg mL^-1^ kanamycin. Subsequently, we diluted the overnight cultures to an initial OD_600_ of 0.01 in M9 culture medium (refer to Table S1 for composition) containing 10 μM MAs(III) and 0.3 mM isopropyl β-D-1- thiogalactopyranoside (IPTG). The cultures were then incubated for 24 h. To monitor cell growth, we measured the optical density at 600 nm at specific time points.

**Design of primer pairs for qPCR to assess the abundance of *Delftia* sp. and *Bacillus* sp. in soil.** To design specific primer pairs for qPCR, we first aligned the draft genomes of DT-2 and BP-3 with other gene sequences from the NCBI database using the BLASTN program. We then identified specific sequence regions of DT-2 and BP-3 and designed primer pairs using Primer Premier 5 software. The specific primer pairs D-F/D-R and B-F/B-R targets the *gcvA* gene (encoding for an HTH-type transcriptional regulator) in DT-2 and the *pepQ* gene (encoding for a putative dipeptidase) in BP-3, respectively. We employed multiple approaches to confirm the specificity of the designed primer pairs. We initially used the ProbeCheck program and the Primer-BLAST program to assess the specificity of the primers. Furthermore, we conducted clone library sequencing and utilized the primers to amplify strains from various genera and species.

To further validate the specificity, we sequenced the PCR amplicons (performed by Biozeron Co., Ltd., Shanghai, China) generated by the D-F/D-R and B-F/B-R primer pairs, using soil DNA as the template. For each pair of primers, we obtained more than 50,000 sequences. The analysis of these sequences revealed that 91% and 93% of the identical sequences corresponded to DT-2 and BP-3, respectively. Moreover, more than 99% of the sequences exhibited over 97% similarity with DT-2 and BP-3 sequences. These highly similar sequences were identified as *Delftia tsuruhatensis*, closely related to DT-2, and *Bacillus pseudomycoides*, closely related to BP-3 (Figure S2). Importantly, the primer pairs failed to amplify CT-33, BT-39, PN-5, *E. coli*, *Arsenicibacter rosenii*, and several other species of *Bacillus*, such as *Bacillus subtilis*, *Bacillus siamensis*, and *Bacillus megaterium*. These results demonstrate the robust specificity of the D-F/D-R and B-F/B-R primer pairs for *Delftia* sp. (including DT-2 and its close relatives) and *Bacillus* sp. (including BP-3 and its close relatives), respectively.

To construct the standard plasmids, we obtained genomic DNA from strains DT-2 and BP-3 and used it as the template for PCR. We generated gene fragments of 16S rRNA, *gcvA*, and *pepQ* by PCR using specific primer pairs (16S-F/16S-R, D-F/D-R, and B-F/B-R) (Table S6) and ligated them into the pEASY®-T3 vector by TA cloning to obtain plasmids pEASY-16S rRNA, pEASY-*gcvA*, and pEASY-*pepQ*. We quantified the DNA concentrations of the plasmids and calculated the copy numbers as described elsewhere [1]. To generate standard curves, we performed 10-fold serial dilutions of the plasmids and utilized these dilutions as templates for PCR amplification. By analyzing the standard curves, we determined the PCR amplification efficiency for each target and confirmed that all amplifications fell within the range of 90-110% efficiency.

**Table S1.** The compositions of the culture media used in this study. For solid media, 2% agar was added for solidification.

| ST10^-1^  medium [8] | tryptone 0.5 g L^-1^, yeast extract 0.05 g L^-1^, D-glucose 5 g L^-1^ (pH = 7.0) |
| --- | --- |
| LB  medium [8] | tryptone 10 g L^-1^, yeast extract 5 g L^-1^, NaCl 10 g L^-1^ |
| M9  medium | Na_2_HPO_4_.7H_2_O 12.8 g L^-1^, KH_2_PO_4_ 3 g L^-1^, NaCl 0.5 g L^-1^, NH_4_Cl 1 g L^-1^, D-glucose 4 g L^-1^ |

**Table S2.** The primers used for the identification of different strains in this study.

| Primer | Sequence |
| --- | --- |
| 27F | AGAGTTTGATCCTGGCTCAG |
| 1492R | TACGACTTAACCCCAATCGC |

**Table S3.** The primers used for qPCR-based abundance measurements of *Delftia* sp. DT-2 and *Bacillus* sp. BP-3.

| Primer | Sequence |
| --- | --- |
| *arsI*-F | GACCGACGATGCGGAGGAAT |
| *arsI*-R | ATGCTGCCCAGCGTATGAAA |
| *gerM*-F | GAGTGGCGGAGGATTTGATT |
| *gerM*-R | TTTGAAACGGGAAGGTGTTC |

**Table S4.** The primers used for RT-qPCR-based gene expression analyses of *BparsM*.

| Primer | Sequence |
| --- | --- |
| 16S rRNA-F | TAGATACCCTGGTAGTCCACGCC |
| 16S rRNA-R | CCCGTCAATTCCTTTGAGTTTCA |
| *arsM*-F | CGGAGGATTTGATTGCTTCTTAG |
| *arsM*-R | AGCACGAGCTTTACTTACCATTT |

**Table S5.** The primers used to amplify the *BparsM* gene from genomic DNA of BP-3 for the construction of plasmid pET29a-*BparsM*.

| Primer | Sequence |
| --- | --- |
| *BparsM*-F | GGAATTCCATATGAAAGAACTTGAAAAAGATCAAAT |
| *BparsM*-R | CCGCTCGAGTGATGGATAGACTGATTTTGGTT |

**Table S6.** The primers used for qPCR-based abundance measurements of *Delftia* sp., *Bacillus* sp., and total bacterial 16S rRNA genes in soils.

| Primer | Sequence |
| --- | --- |
| 16S-F | CGGTGAATACGTTCYCGG |
| 16S-R | GGWTACCTTGTTACGACTT |
| D-F | CGATGTGGTGGCCGAGCCCTTTCTGCAAGAG |
| D-R | GCACGCCGGTCAGTTCGCTCCAATGCCGCCA |
| B-F | GCTCGTTCTGTAATCGCAGATGCTGGCTACG |
| B-R | ATGCCTTCACGAAGAGGTGAGTTGTTACCTT |


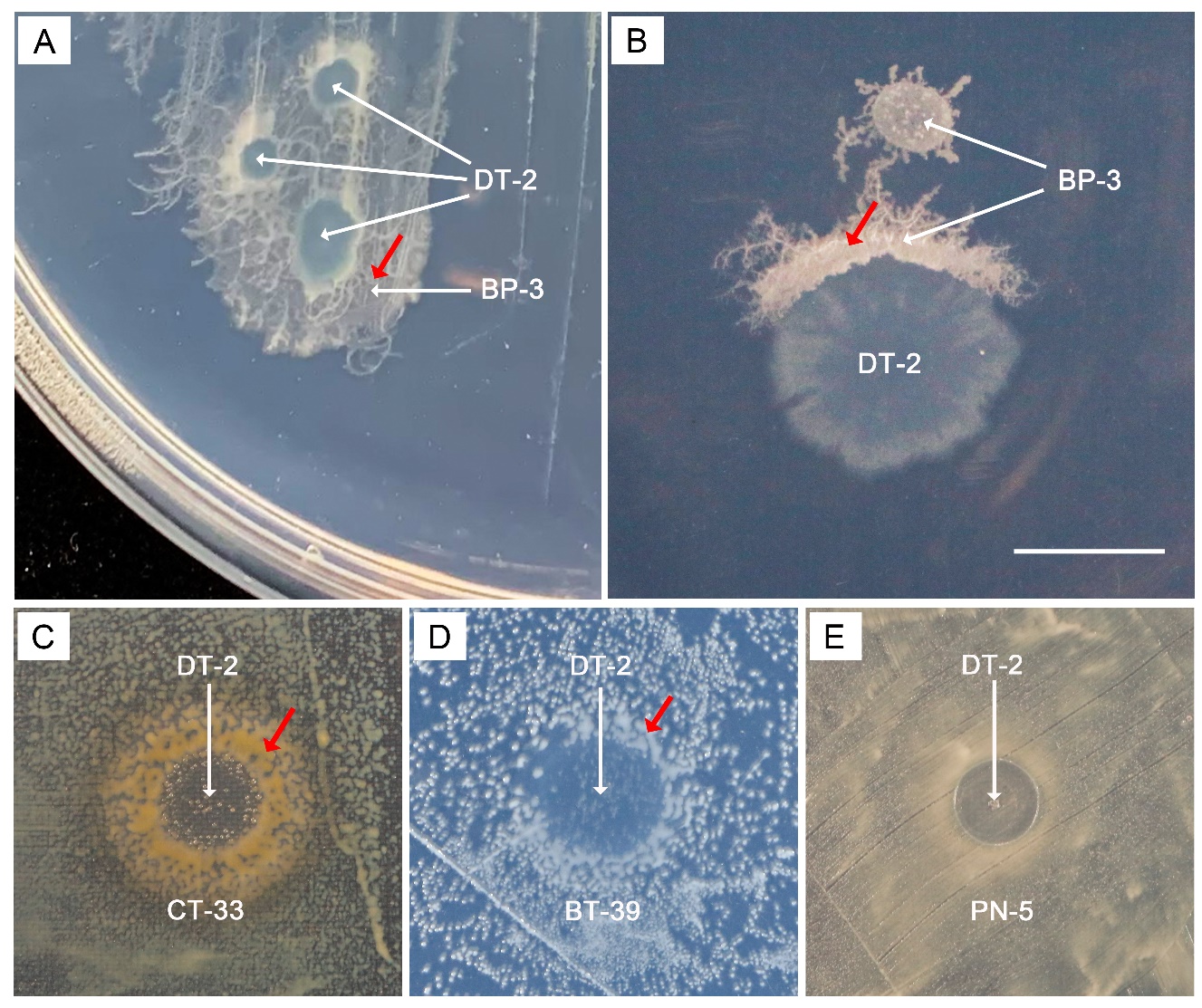


**Figure S1.** Growth of different strains together on agar media. **A**, **B** *Delftia* sp. DT-2 and *Bacillus* sp. BP-3. **C** *Delftia* sp. DT-2 and *Chryseobacterium* sp. CT-33. **D** *Delftia* sp. DT-2 and *Bacillus* sp. BT-39. **E** *Delftia* sp. DT-2 and *Paenarthrobacter* sp. PN-5. It should be noted that during growth, both *Delftia* sp. DT-2 and *Bacillus* sp. BP-3 strains often exhibit physical contact with each other. Regions highlighted by red arrows demonstrated higher bacterial growth compared to the other areas. The scale bar represents 1.0 cm.


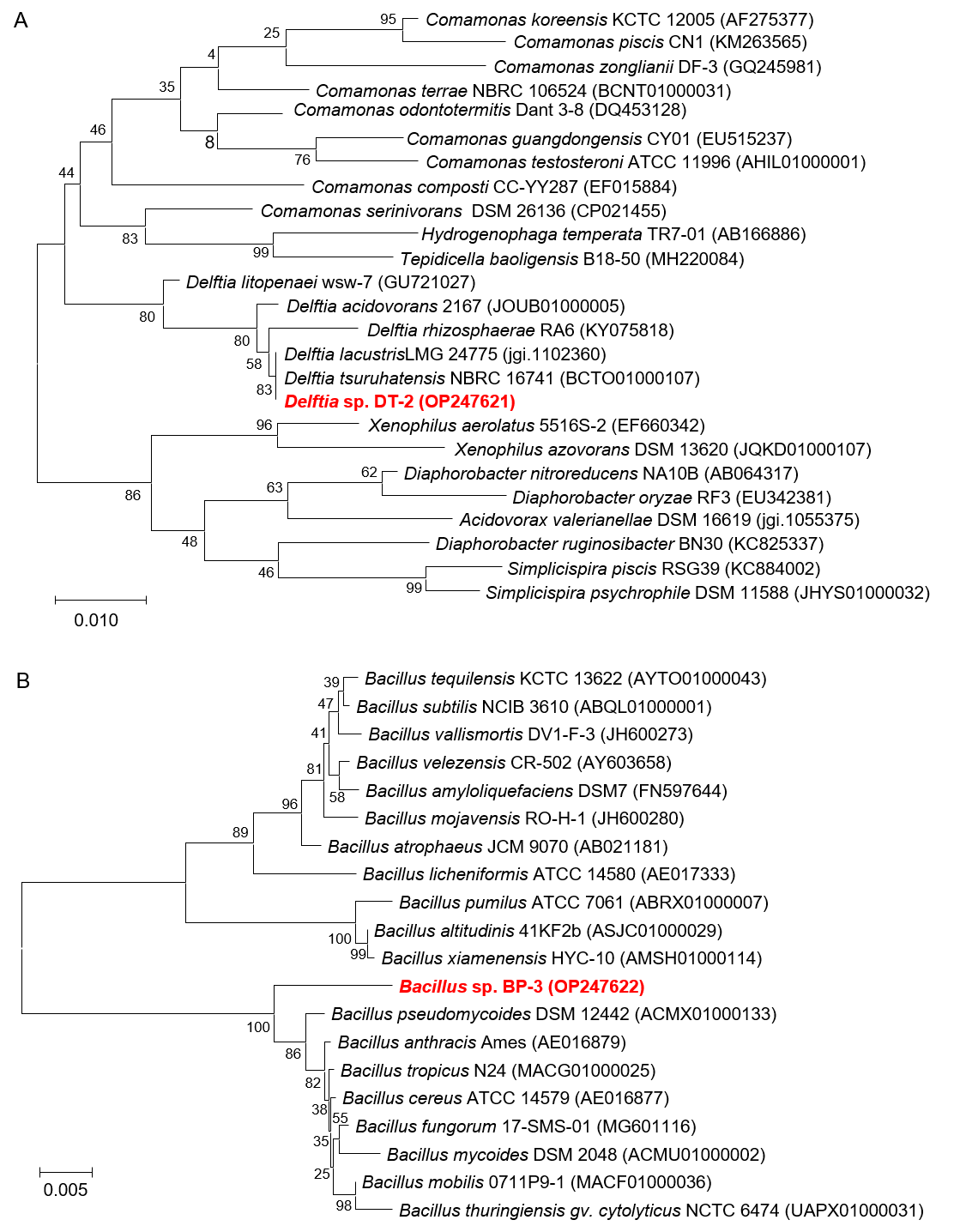


**Figure S2.** Neighbor-joining phylogenetic trees based on 16S rRNA gene sequences showing the evolutionary relationships of *Delftia* sp. DT-2 (**A**) and *Bacillus* sp. BP-3 (**B**) with representative members of the genus.


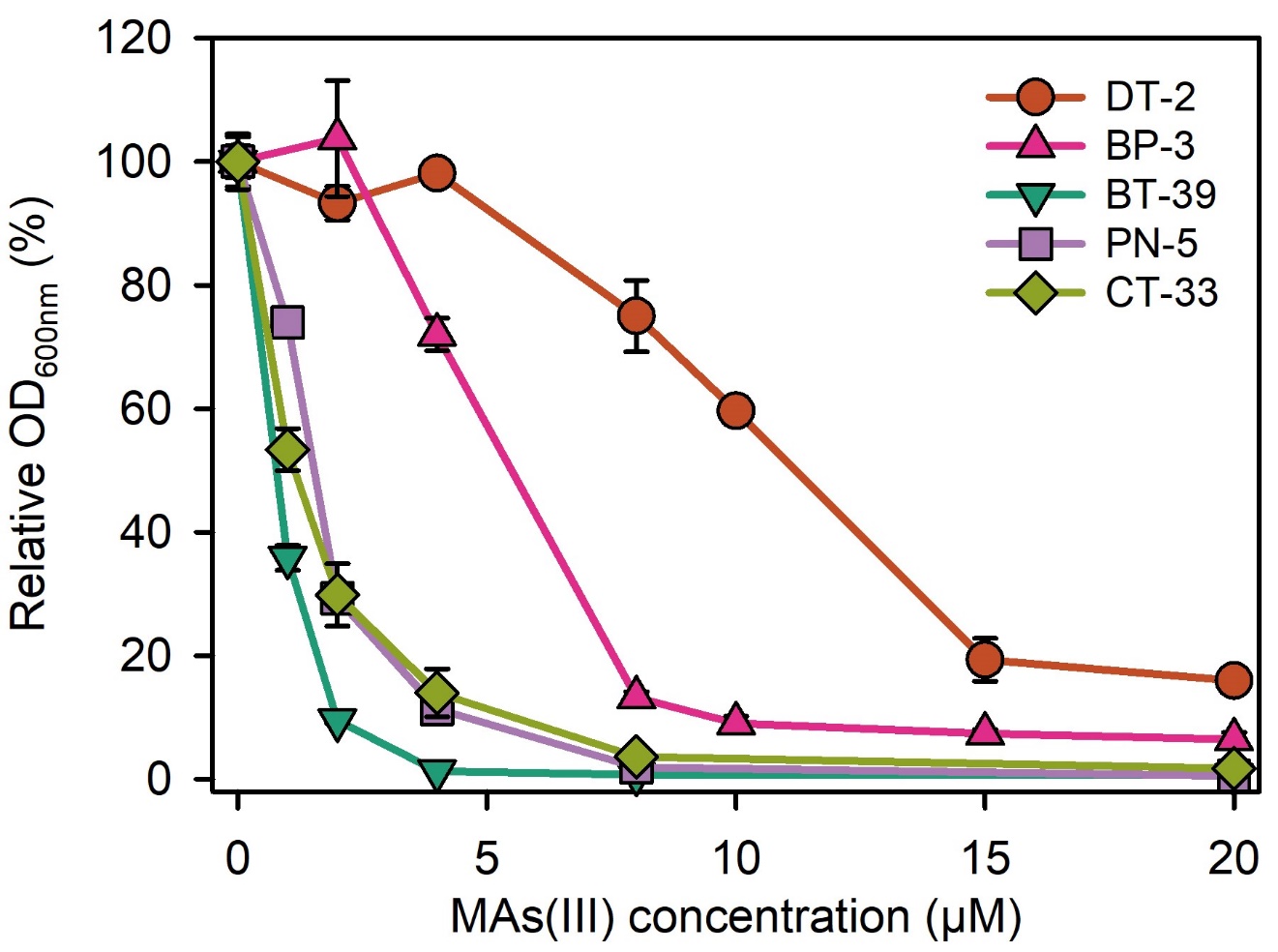


**Figure S3.** Growth of DT-2, BP-3, BT-39, PN-5, and CT-33 in ST10^-1^ media containing varying concentrations of MAs(III). The data are presented as the mean ± standard error (n = 3).


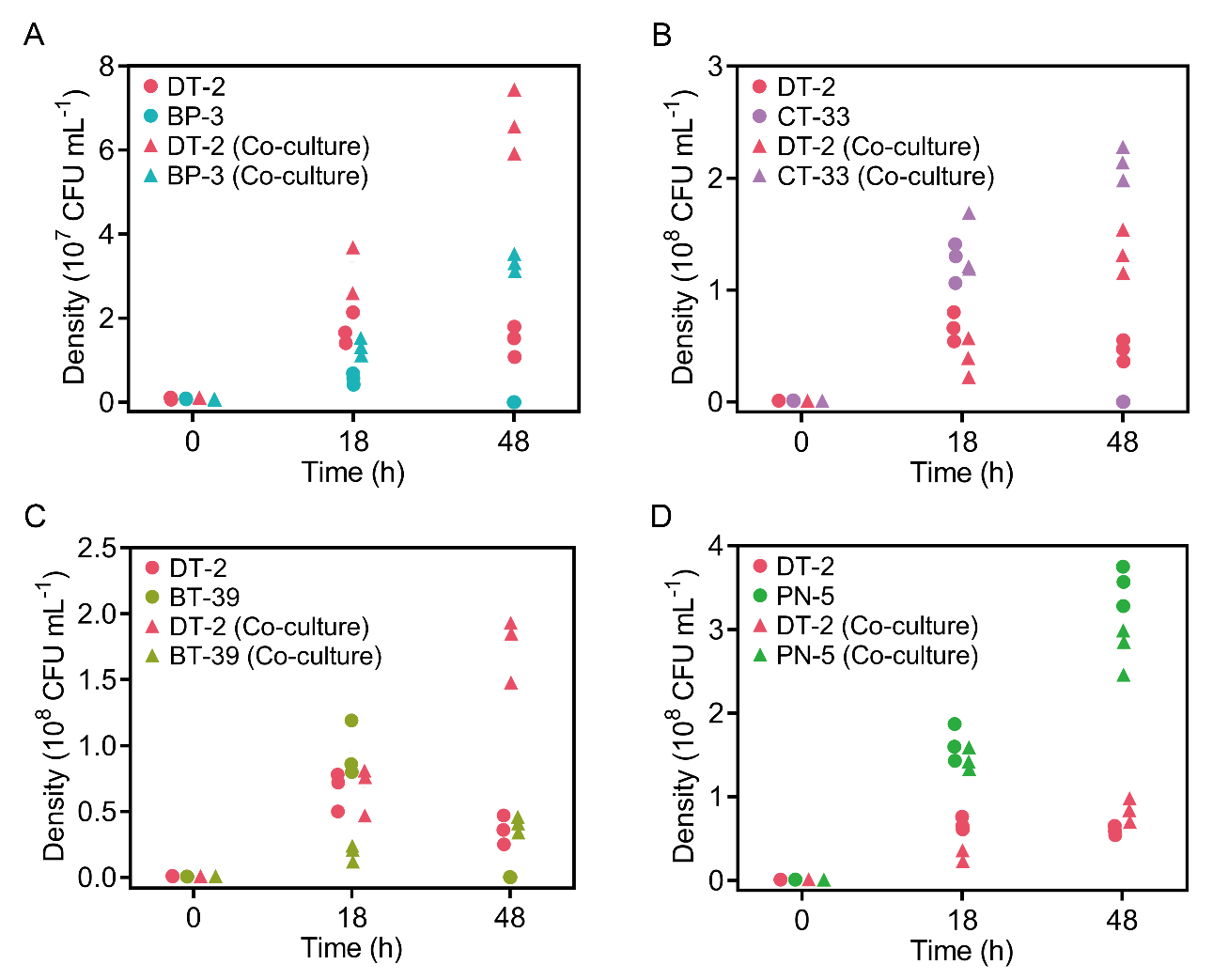


**Figure S4.** Growth of strains in mono- or co-cultures. **A** *Delftia* sp. DT-2 and *Bacillus* sp. BP-3. **B** *Delftia* sp. DT-2 and *Chryseobacterium* sp. CT-33. **C** *Delftia* sp. DT-2 and *Bacillus* sp. BT-39. **D** *Delftia* sp. DT-2 and *Paenarthrobacter* sp. PN-5. Density of viable cells in the cultures was determined by counting colony-forming units (CFUs).


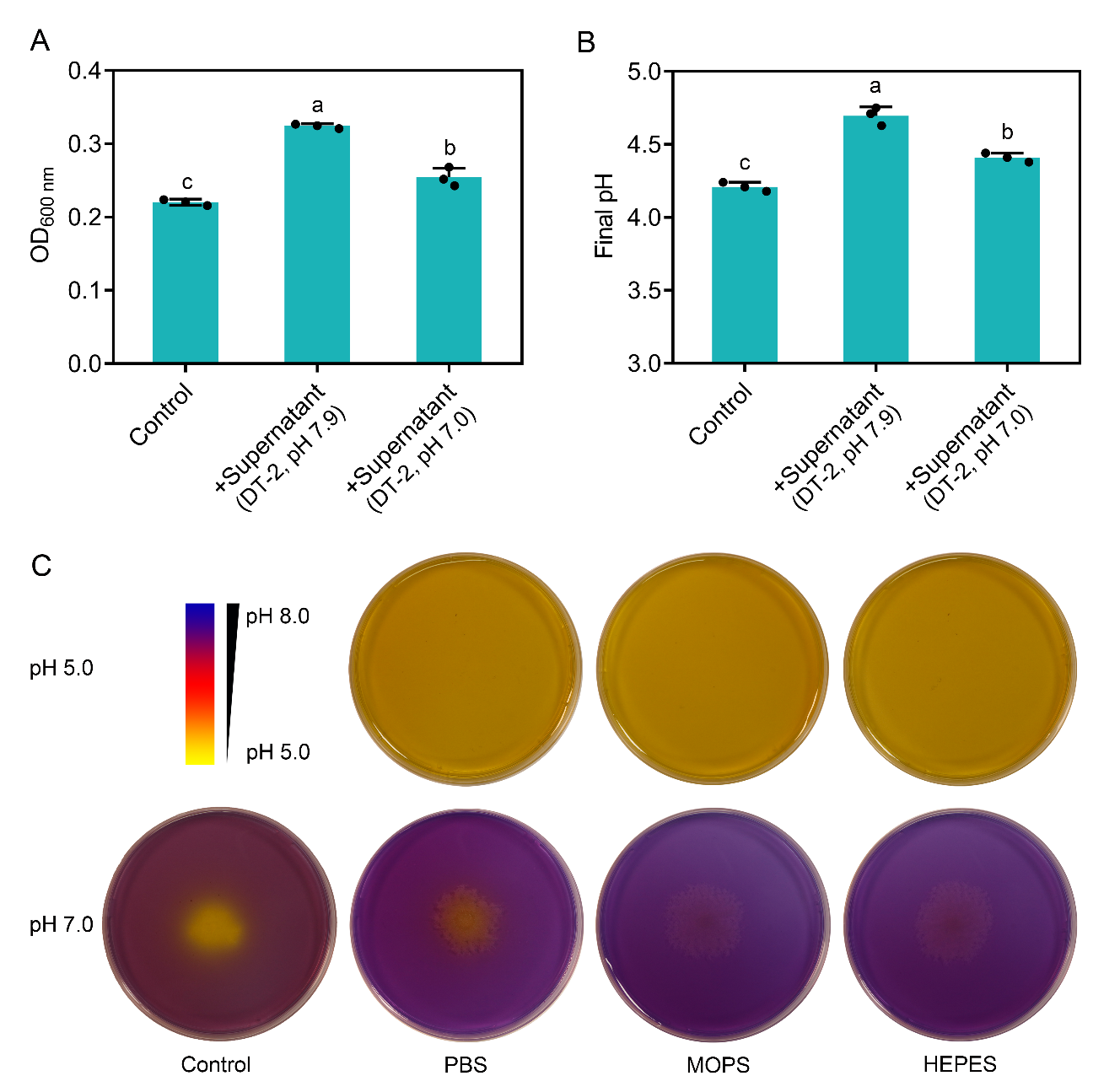


**Figure S5.** Effect of pH on the growth of *Bacillus* sp. BP-3. **A**, **B** The growth of BP-3 (**A**) and the pH (**B**) of the culture medium containing *Delftia* sp. DT-2 culture supernatants (at pH 7.9 or pH 7.0) for two days. A control group consisted of 10 mL fresh ST10^-1^ liquid medium. The data are presented as the mean ± standard error (n = 3). Different letters indicate significant difference among treatments at *p* < 0.05. **C** The growth of BP-3 on ST10^-1^ agar plates (at initial pH 5.0 or 7.0) with various buffers including 50 mM phosphate, HEPES, or MOPS buffers for two days, with bromocresol violet as a pH indicator. The control did not contain any buffer.


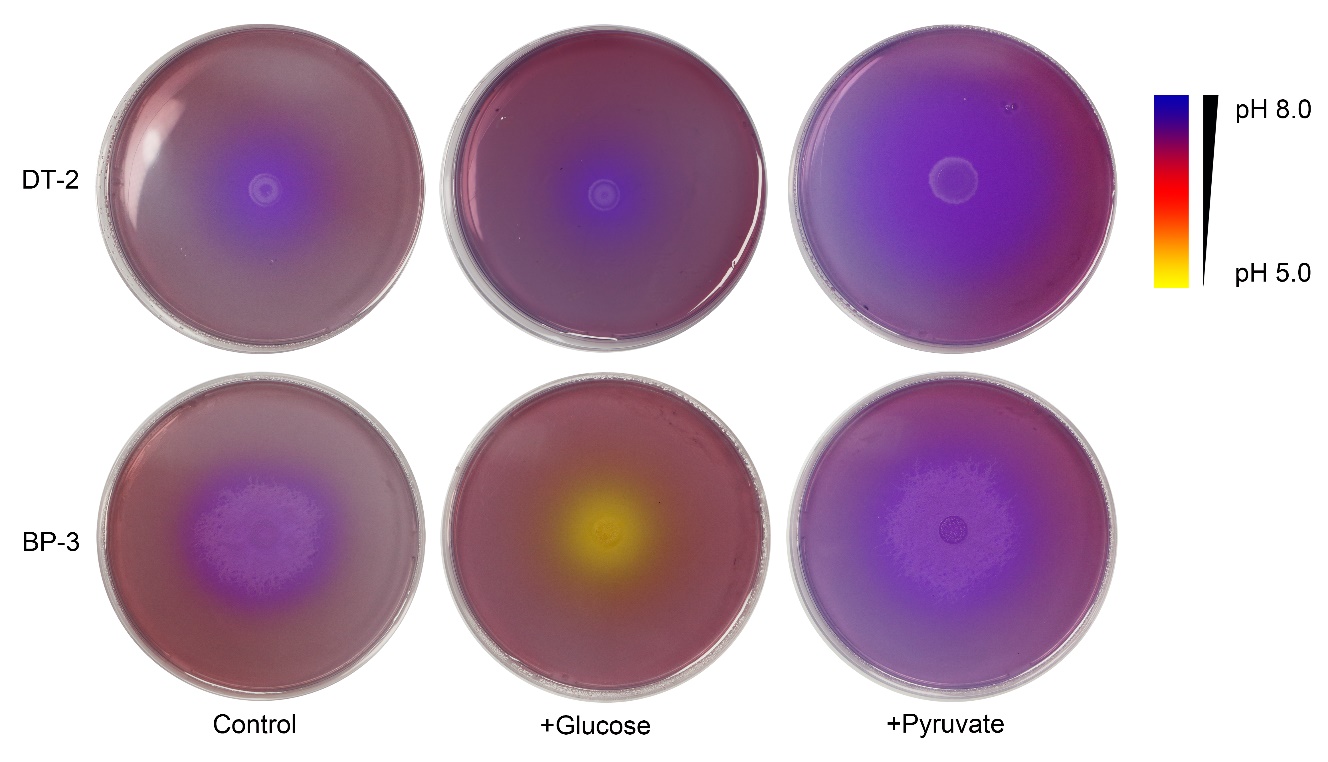


**Figure S6.** Effect of glucose and pyruvate on the growth of *Delftia* sp. DT-2 and *Bacillus* sp. BP-3. The two strains were grown on ST10^-1^ agar plates containing 30 mM glucose or 10 mM pyruvate for two days with bromocresol violet as a pH indicator. The initial pH of the medium was 7.0. The control group did not contain glucose and pyruvate.


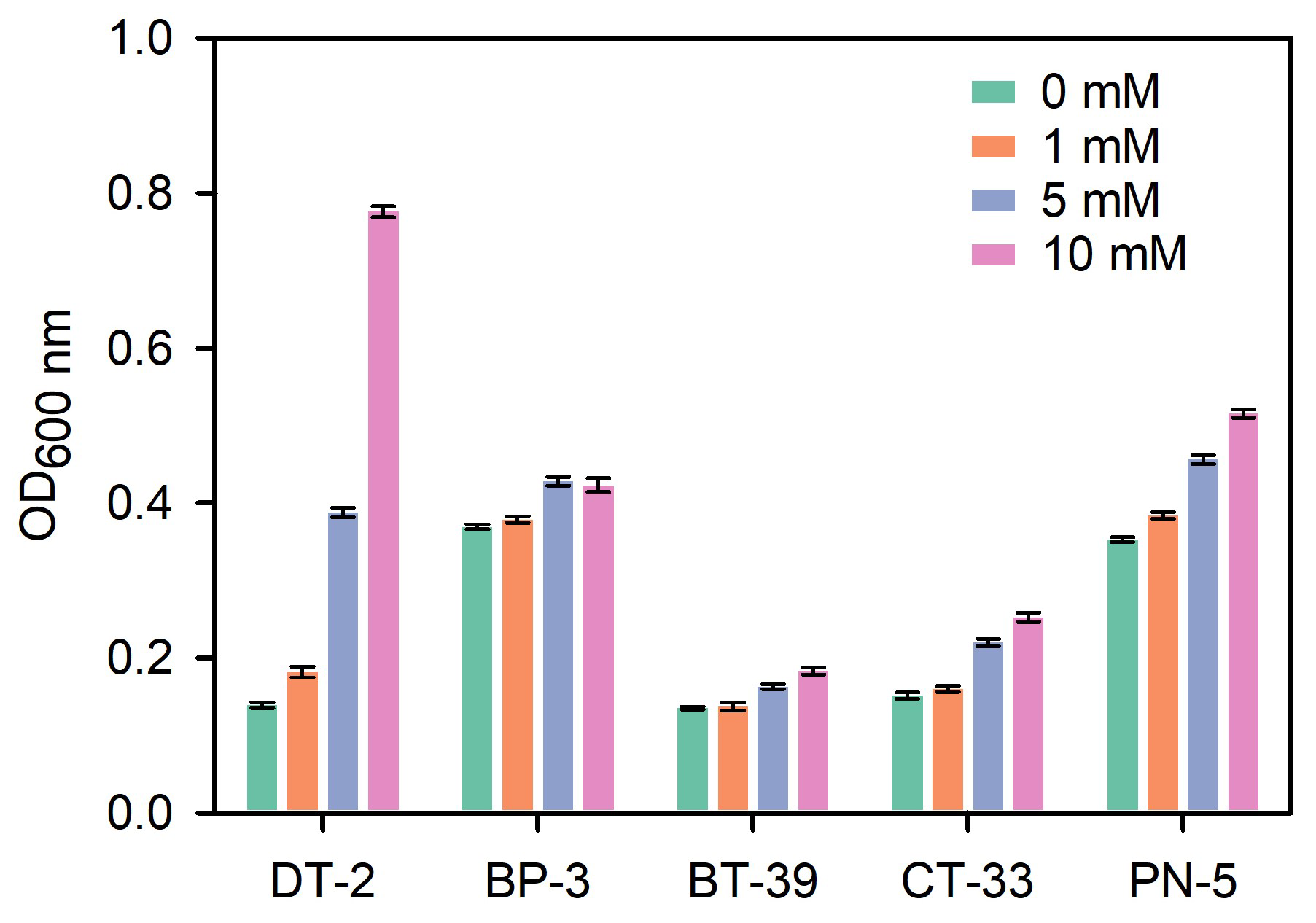


**Figure S7.** Effect of sodium pyruvate on the growth of strains. Strains DT-2, BP-3, BT-39, CT-33, and PN-5 were inoculated separately in ST10^-1^ culture medium (without glucose) supplemented with different concentrations of sodium pyruvate (0, 1, 5, or 10 mM). The initial pH of the medium was 7.0. The cultures were incubated for two days, and the OD_600_ of the cultures were recorded at last. The data are presented as the mean ± standard error (n = 3).


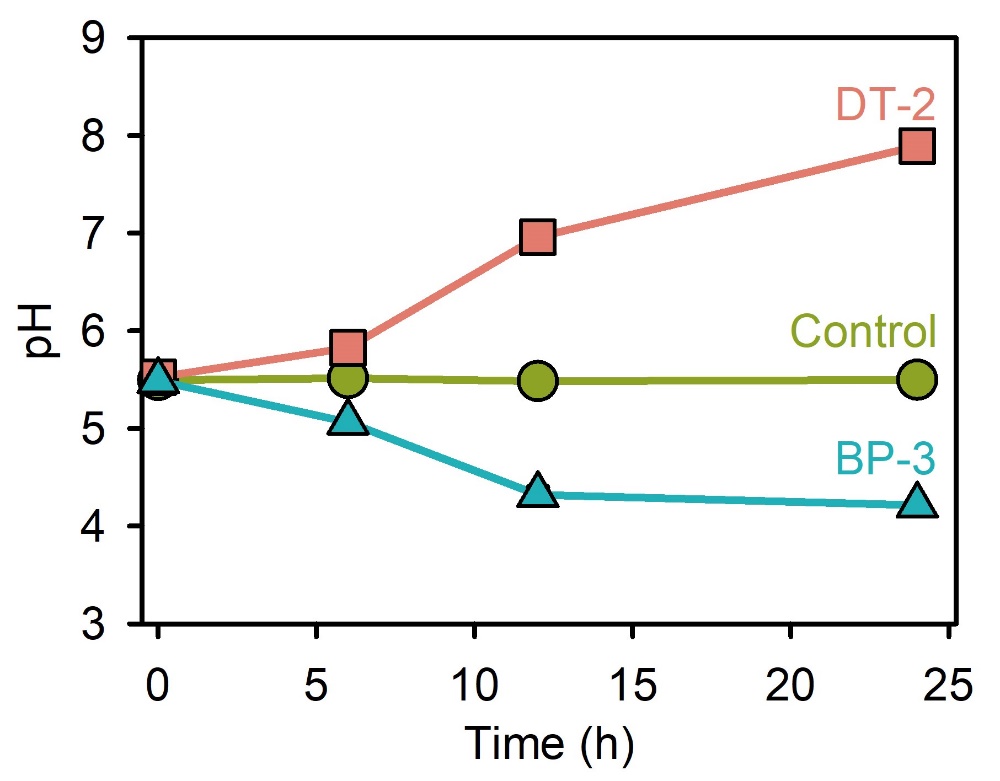


**Figure S8.** The impact of DT-2 and BP-3 on the acidification of the medium caused by exogenously added pyruvic acid. Pyruvic acid was initially introduced into the ST10^-1^ medium to lower the pH to 5.5, after which DT-2 and BP-3 strains were separately inoculated into the culture medium for cultivation. The pH of the culture medium was monitored every 6 h. The control group received no inoculation. The data are presented as the mean ± standard error (n = 3).


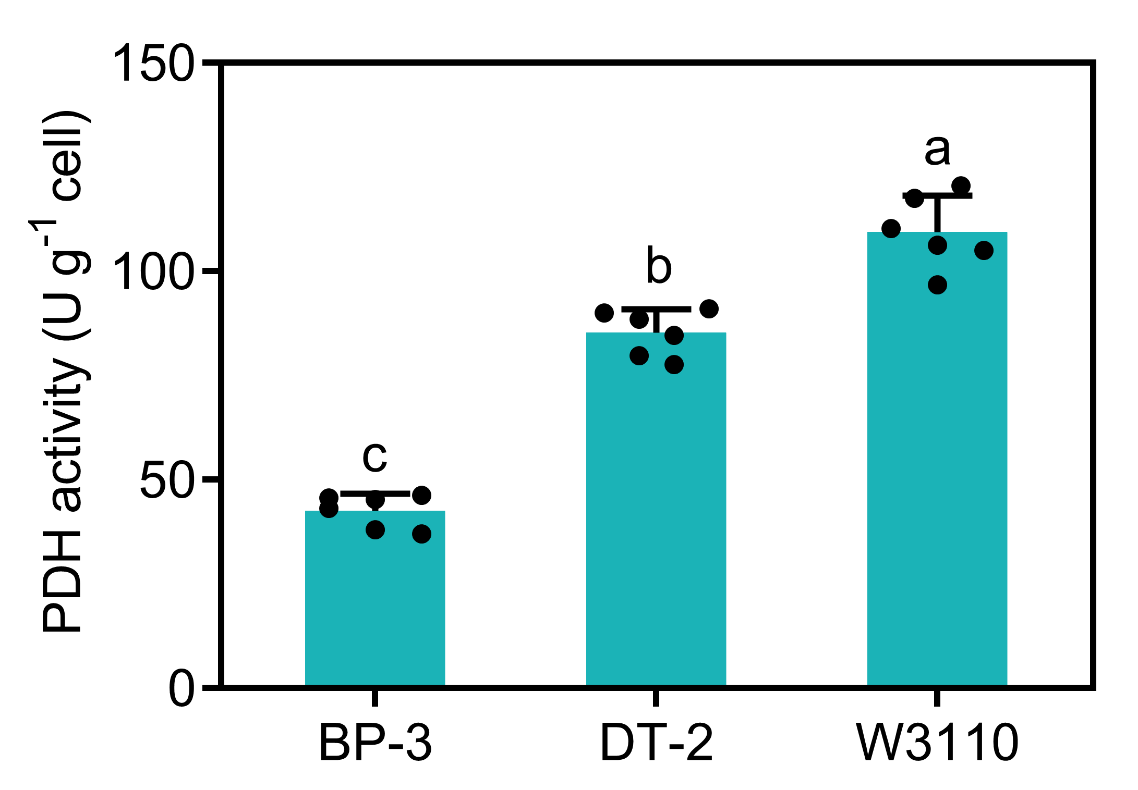


**Figure S9.** Pyruvate dehydrogenase activity of *Bacillus* sp. BP-3, *Delftia* sp. DT-2, and *E.coli* W3110. The three strains were incubated in ST10^-1^ culture medium supplied with 10 mM pyruvate for 12 h. The pyruvate dehydrogenase activity was determined by a PDH activity assay kit based on a colorimetric method. The data are presented as the mean ± standard error (n = 3). Different letters indicate significant difference among treatments at *p* < 0.05.


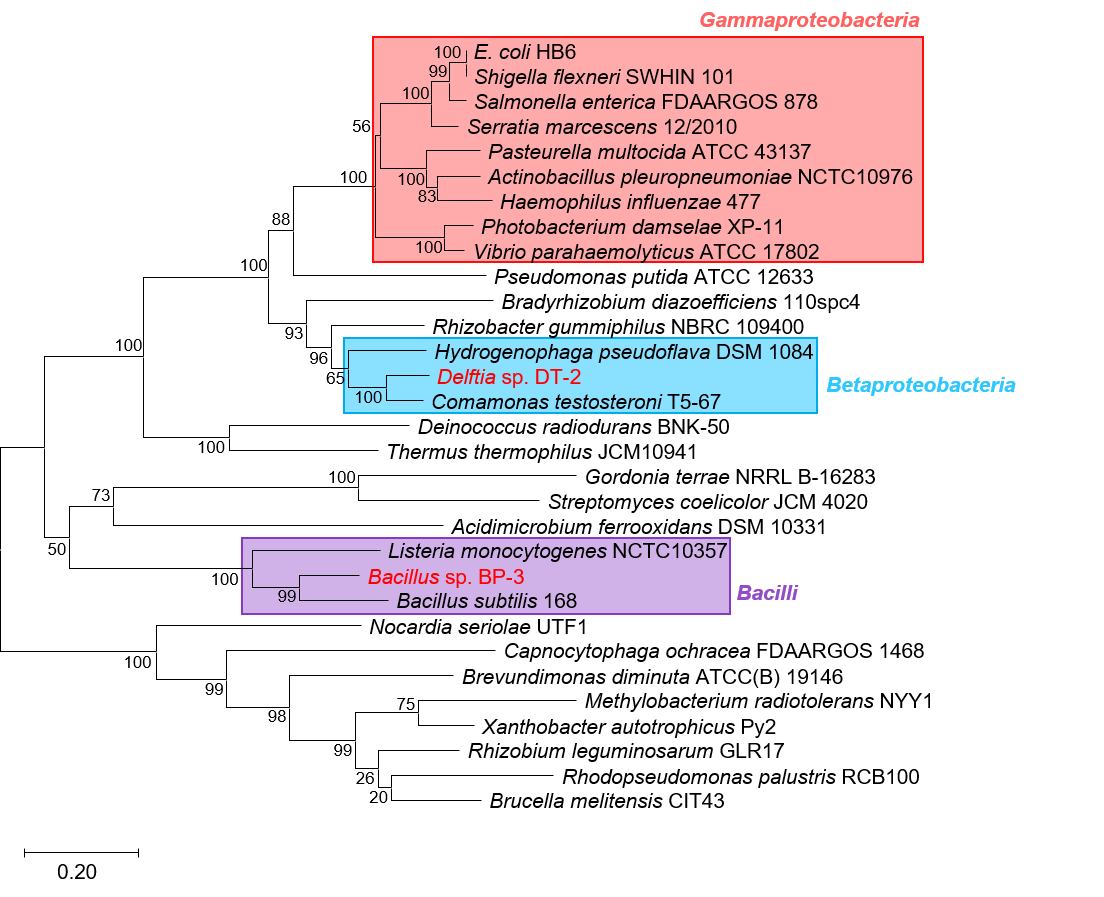


**Figure S10.** Phylogenetic trees of major lineages of dihydrolipoamide acetyltransferase (E2). The evolutionary history was inferred using the Neighbor-Joining method which is based on dihydrolipoamide acetyltransferase protein sequences of *Bacillus* sp. BP-3, *Delftia* sp. DT-2 and 30 other different genera.


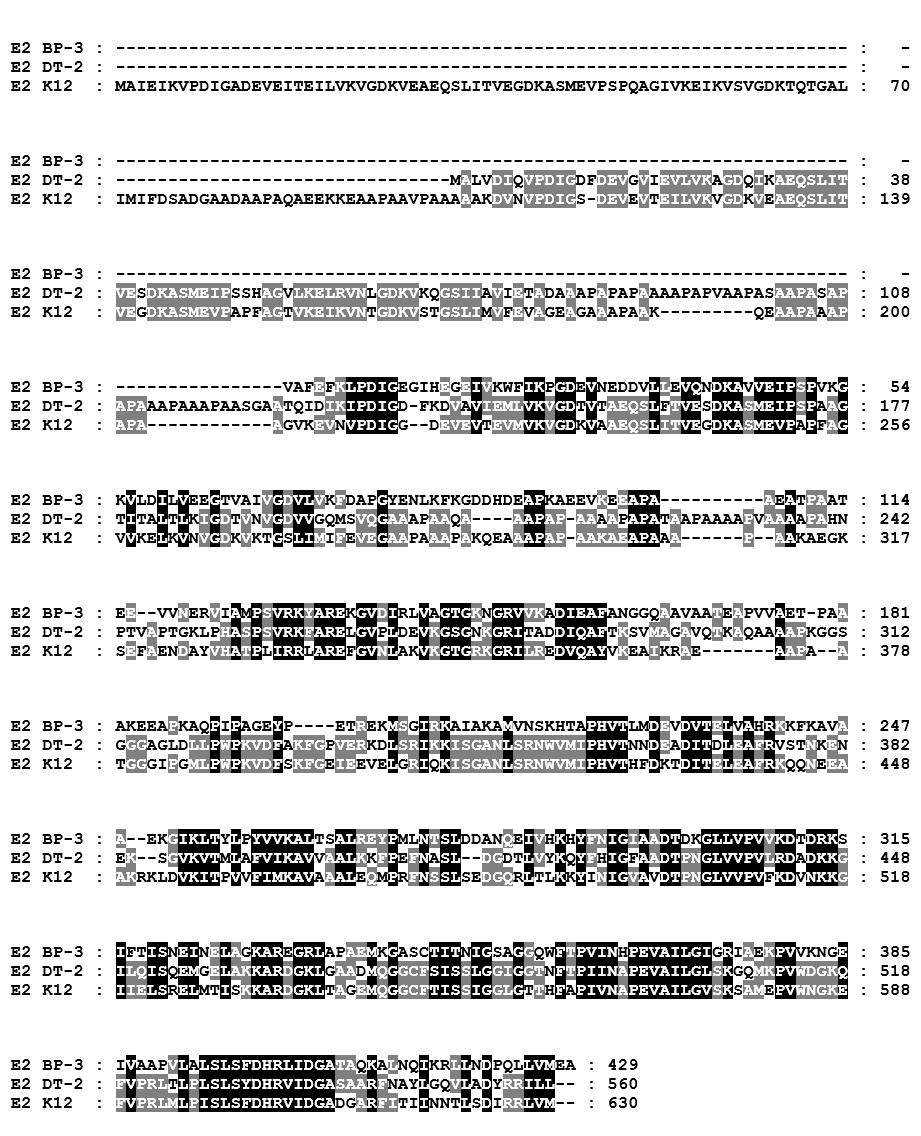


**Figure S11.** Multiple alignment of the dihydrolipoamide acetyltransferase (E2) of pyruvate dehydrogenase multienzyme (PDH) complex orthologs. E2 sequences were compared from *Bacillus* sp. BP-3, *Delftia* sp. DT-2 and *E. coli* K12. Identities are highlighted in black and conservative replacements in gray.


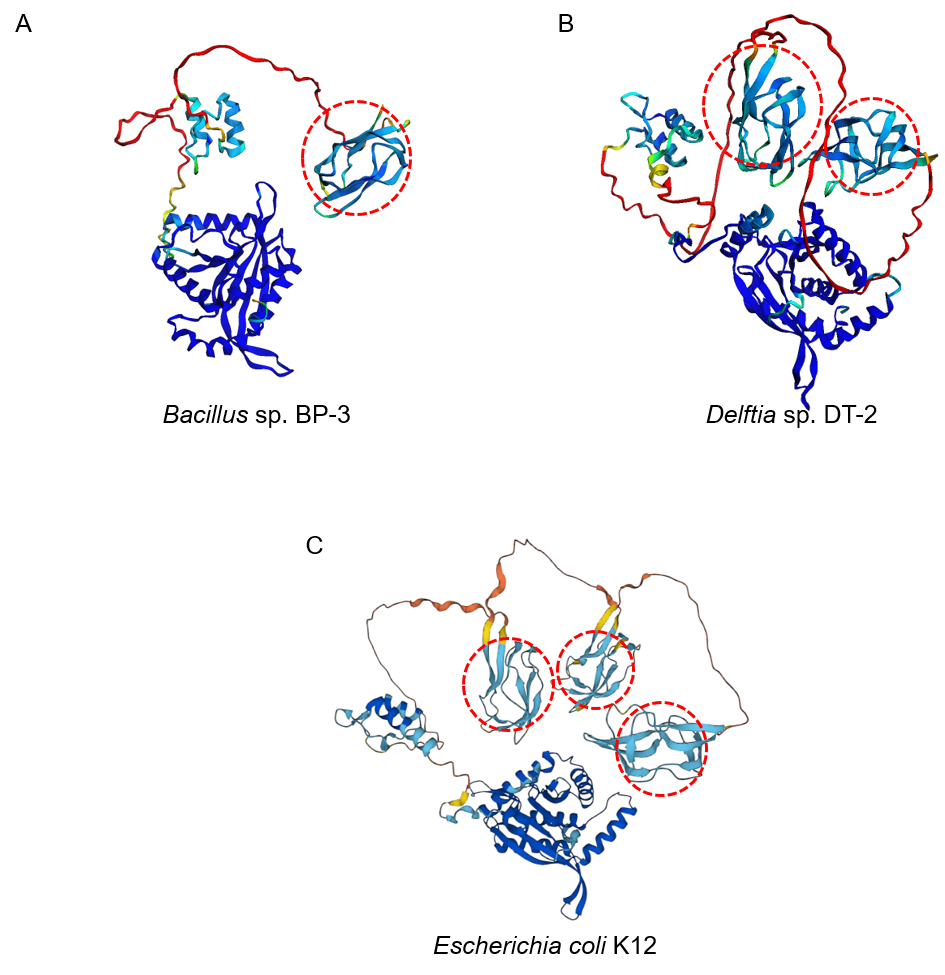


**Figure S12.** Structural prediction of dihydrolipoamide acetyltransferase (E2) of *Bacillus* sp. BP-3 (**A**), *Delftia* sp. DT-2 (**B**), and *E. coli* K12 (**C**). The lipoyl domains are highlighted by the dashed red circles.


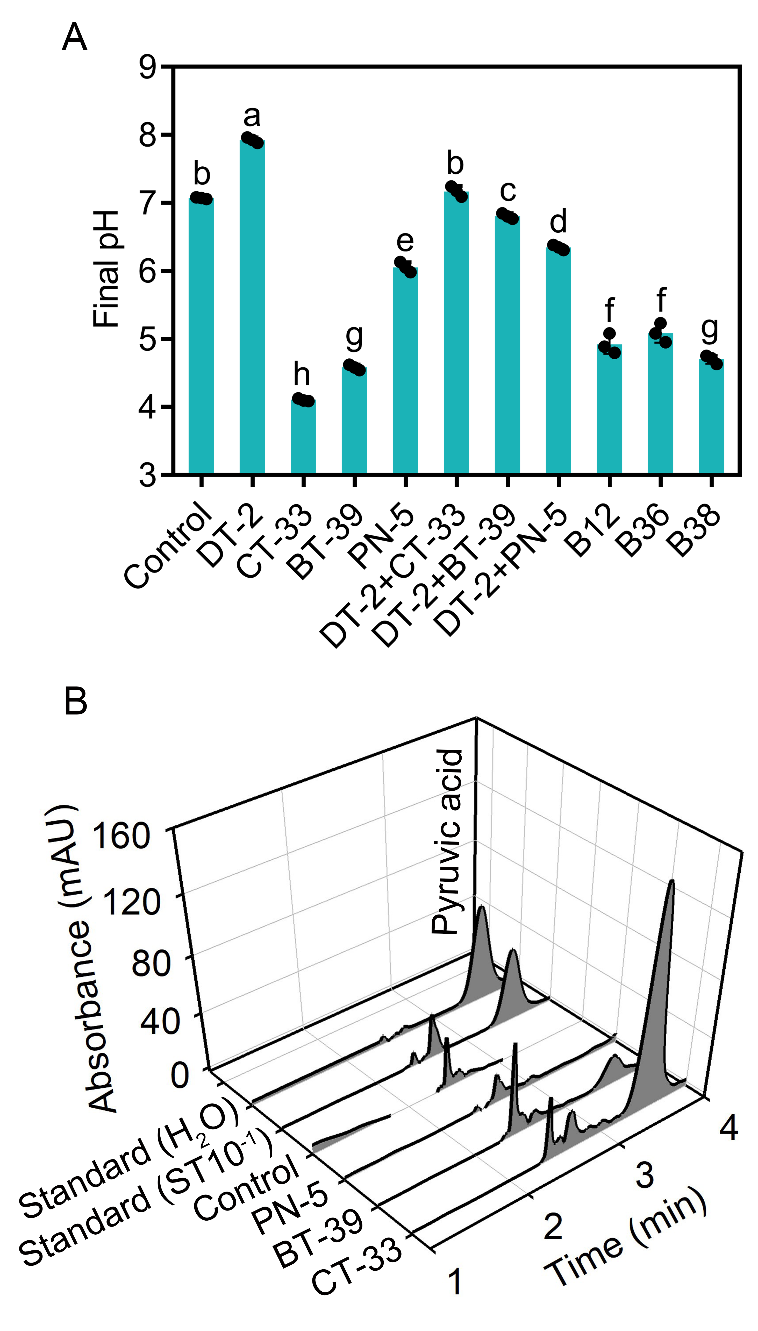


**Figure S13.** The production of pyruvic acid by strains and the acidification of the growth media. **A** Medium pH of DT-2, CT-33, BT39, PN-5, B12, B36, and B38 under mono- and co-culture conditions in ST10^-1^ culture medium. The control was no inoculation treatment. B12, B36, and B38 are all *Bacillus*. The data are presented as the mean ± standard error (n = 3). Different letters indicate significant difference among treatments at *p* < 0.05. **B** Pyruvic acid was produced by strains CT-33, PN-5, and BT-39 in mono-culture. We cultivated these strains in ST10^-1^ medium with 5.0 mM D-glucose for two days.


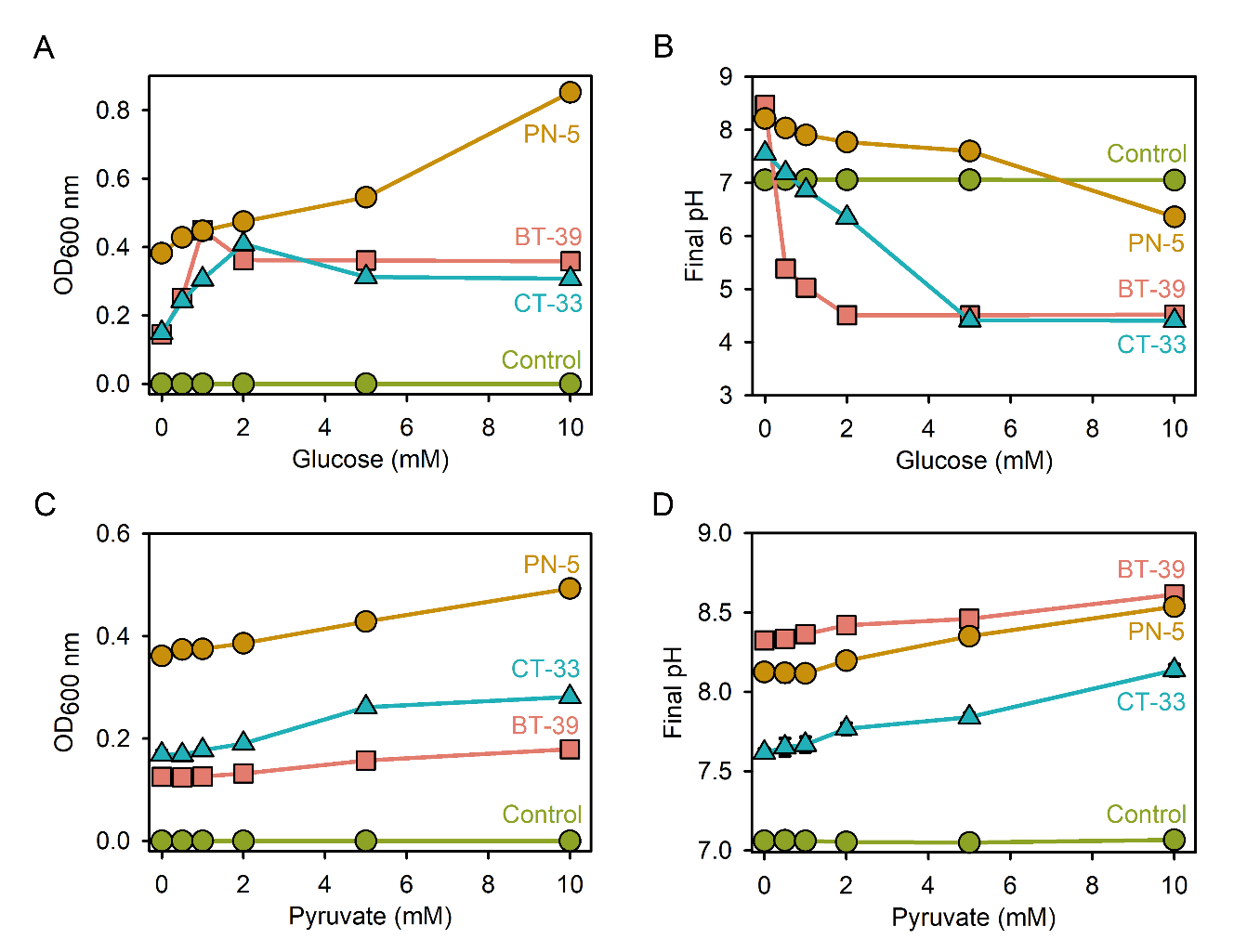


**Figure S14.** Utilization of glucose and pyruvate by the competitors of strains in mono-culture. **A**, **B** Effects of glucose on bacterial growth (**A**) and pH of culture medium (**B**). **C**, **D** Effects of pyruvate on bacterial growth (**C**) and pH of culture medium (**D**). Strains BT-39, CT-33, and PN-5 were inoculated separately in ST10^-1^ culture medium (without glucose) supplemented with different concentrations of glucose or pyruvate (0, 0.5, 1, 2, 5, or 10 mM). The cultures were incubated for two days, and the OD_600_ and pH of the cultures were recorded at last. The control received no inoculation. The data are presented as the mean ± standard error (n = 3).


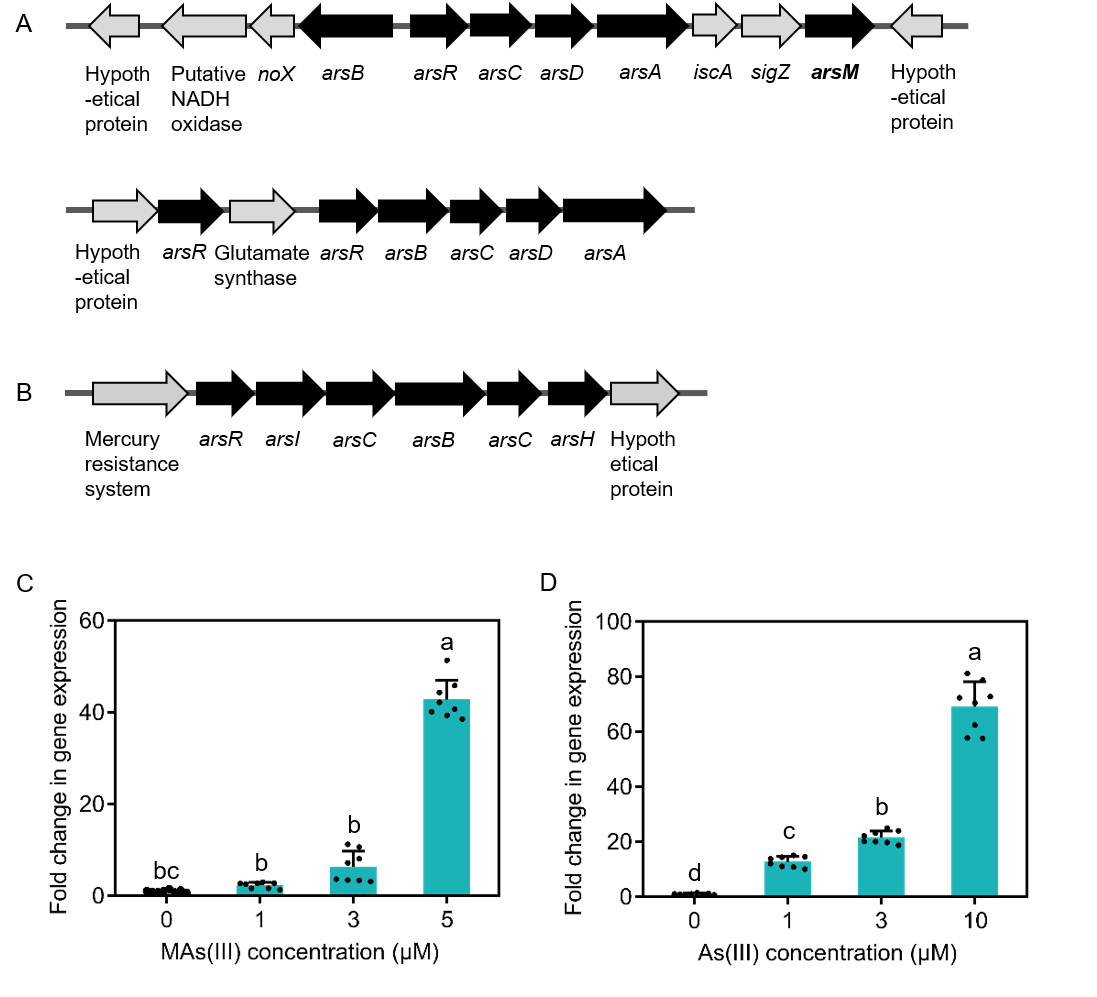


**Figure S15.** Arsenic-related genes and transcription analysis in *Bacillus* sp. BP-3 and *Delftia* sp. DT-2. **A** The *ars* operon and *arsM* gene of *Bacillus* sp. BP-3. **B** The *ars* operon of *Delftia* sp. DT-2. **C**, **D** The transcription level of *BparsM* measured by RT-qPCR in the presence of different concentrations of MAs(III) (0, 1, 3, or 5 μM) (**C**) or As(III) (0, 1, 3, and 10 μM) (**D**). The data are presented as the mean ± standard error (n = 8). Different letters indicate significant difference among treatments at *p* < 0.05.


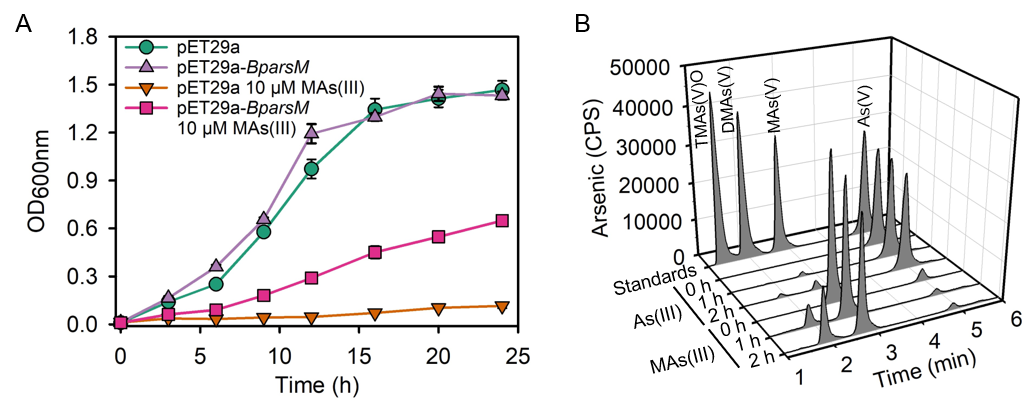


**Figure S16.** BpArsM confers MAs(III) resistance through MAs(III) methylation in strain *E. coli* AW3110. **A** Strain AW3110 bearing either plasmid pET29a-*BparsM* or pET29a was grown in M9 minimal medium with 0.3 mM IPTG with or without the presence of MAs(III) for 24 h. The data are presented as the mean ± standard error (n = 3). **B** Methylation of MAs(III) or As(III) by BpArsM in vitro. The reaction mixture containing 2.0 μM purified BpArsM protein, 1.0 mM SAM, and 8.0 mM GSH, was incubated in the presence of 10 μM MAs(III) or As(III) at 30˚C for the indicated times. All reactions were terminated by adding 6% (V/V) H_2_O_2_ and various arsenic species were analyzed by HPLC-ICP-MS.


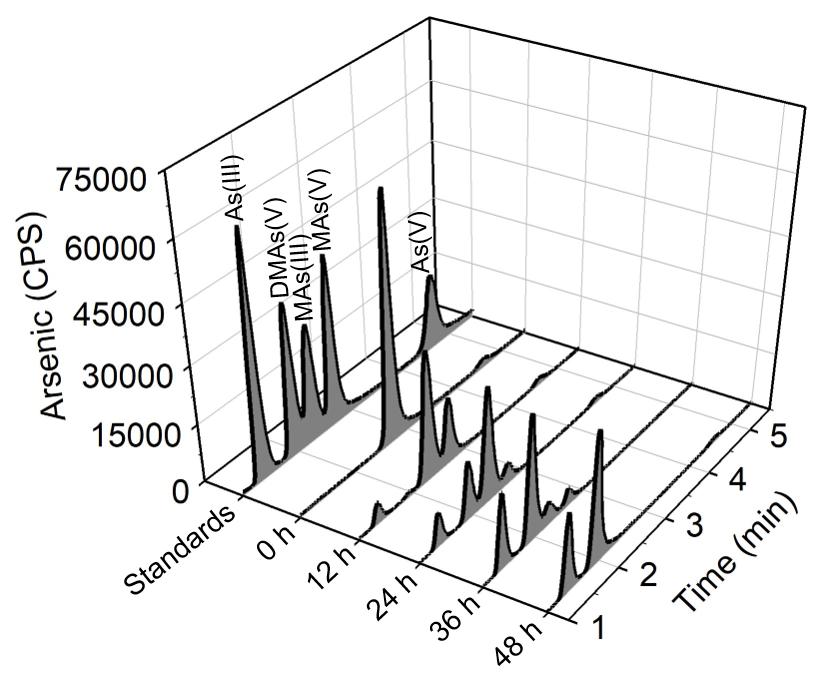


**Figure S17.** Time course of MAs(V) reduction and methylation in the co-culture media of *Delftia* sp. DT-2 and *Bacillus* sp. BP-3. Arsenic species in the reaction systems were analyzed by HPLC-ICP-MS.


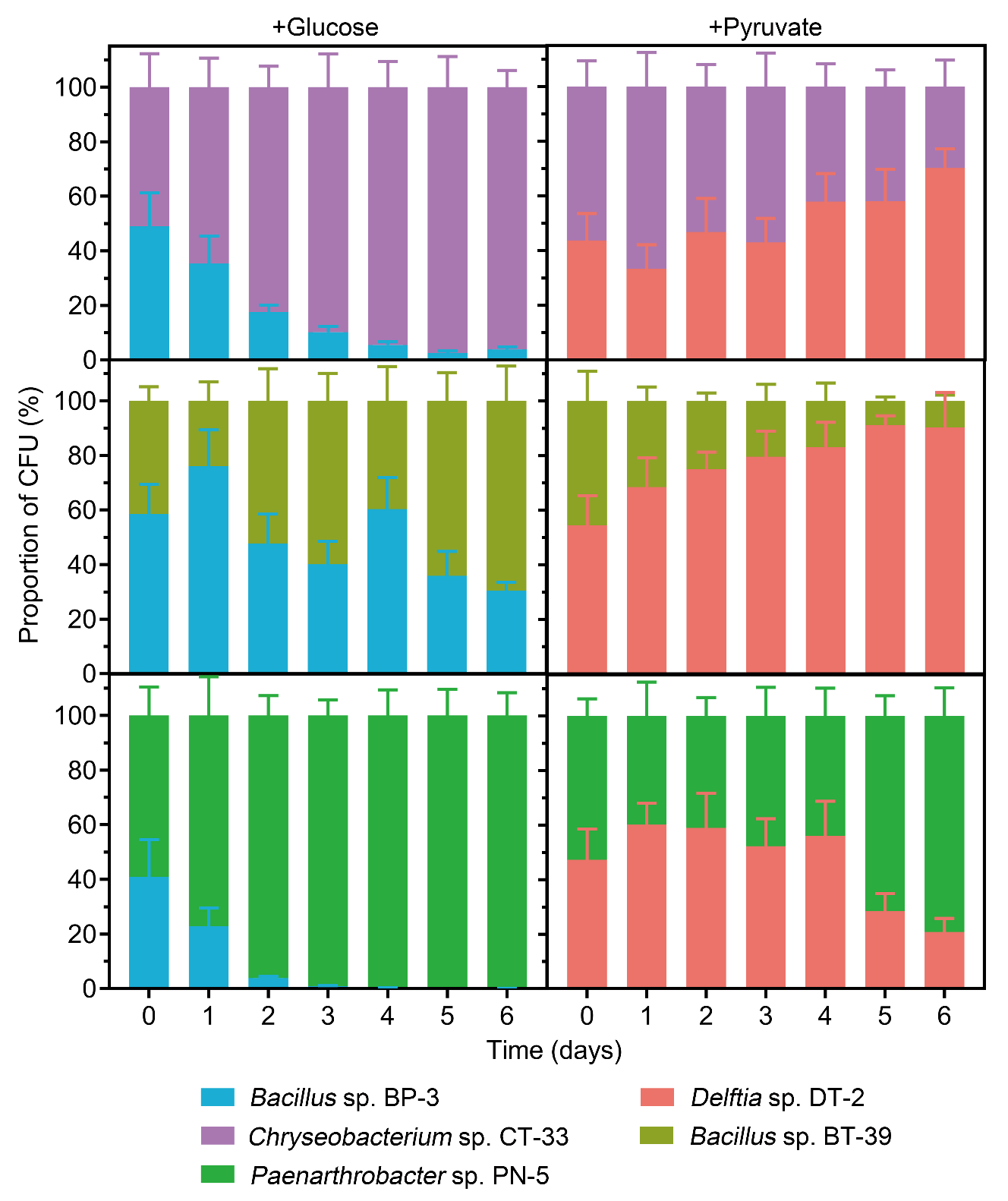


**Figure S18.** The growth of communities consisting of two strains in the growth media supplemented with glucose or pyruvate. BP-3 or DT-2 was co-cultured with one of CT-33, BT-39, and PN-5 in ST10^-1^ liquid media (without glucose) with 30 mM glucose or 10 mM pyruvate and then incubated for one day. The co-cultures were serially transferred every day for six days. The data are presented as the mean ± standard error (n = 3).


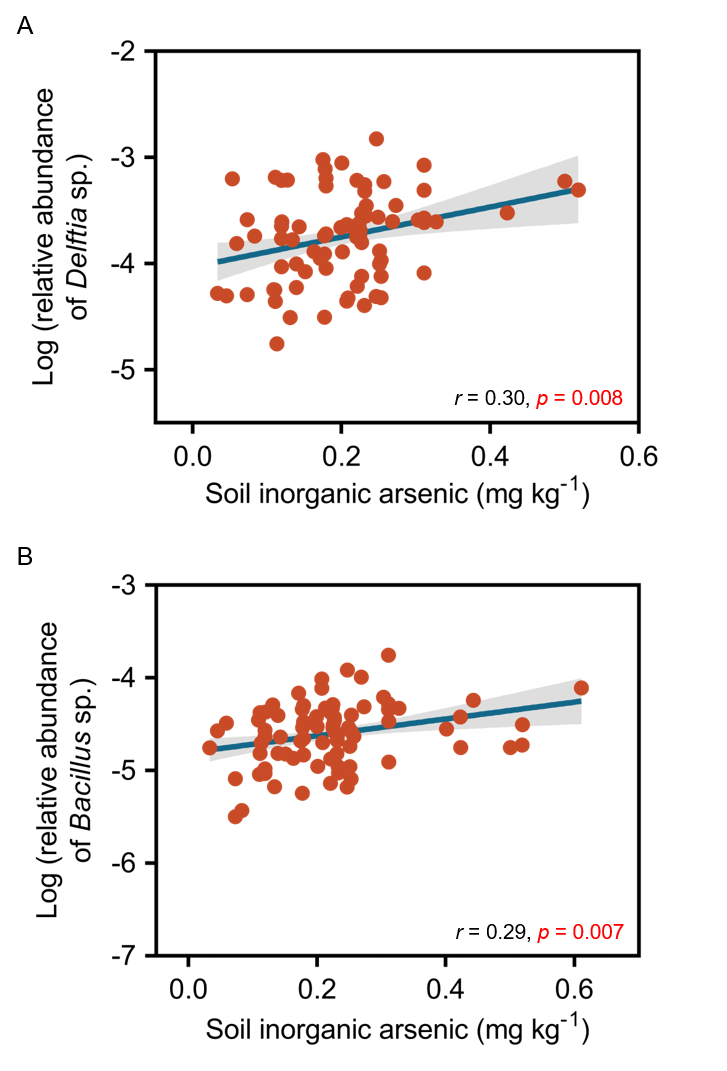


**Figure S19.** The relative abundances of *Delftia* sp. (**A**) and *Bacillus* sp. (**B**) as a function of soil inorganic arsenic concentrations (iAs).


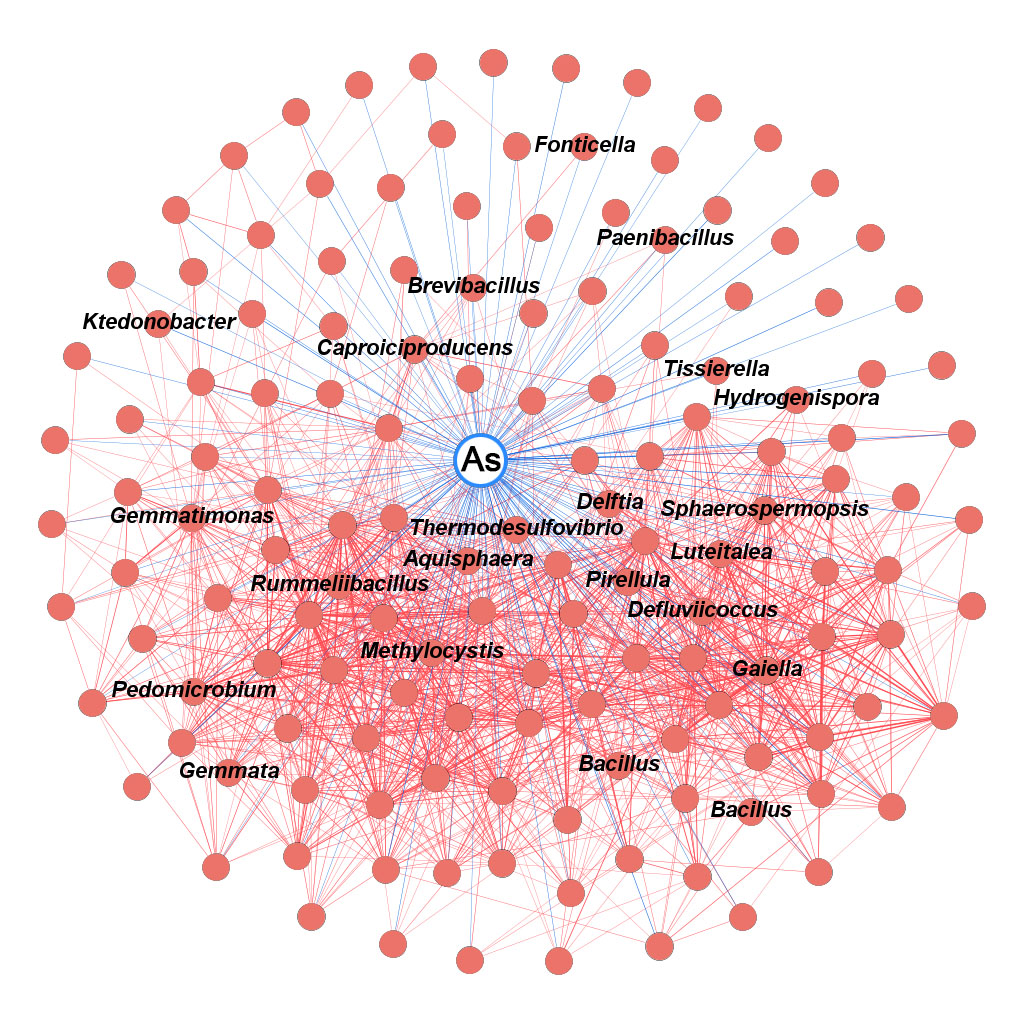


**Figure S20.** Correlations of the relative abundance among microorganisms and the correlations between the relative abundance and soil arsenic concentration based on the 16S rRNA gene data from 103 soils. The line thickness is proportional to the degree of correlation and only significantly positive correlations are presented (*R* > 0.3, *p* < 0.01). All genus names refer to a species of the genus.


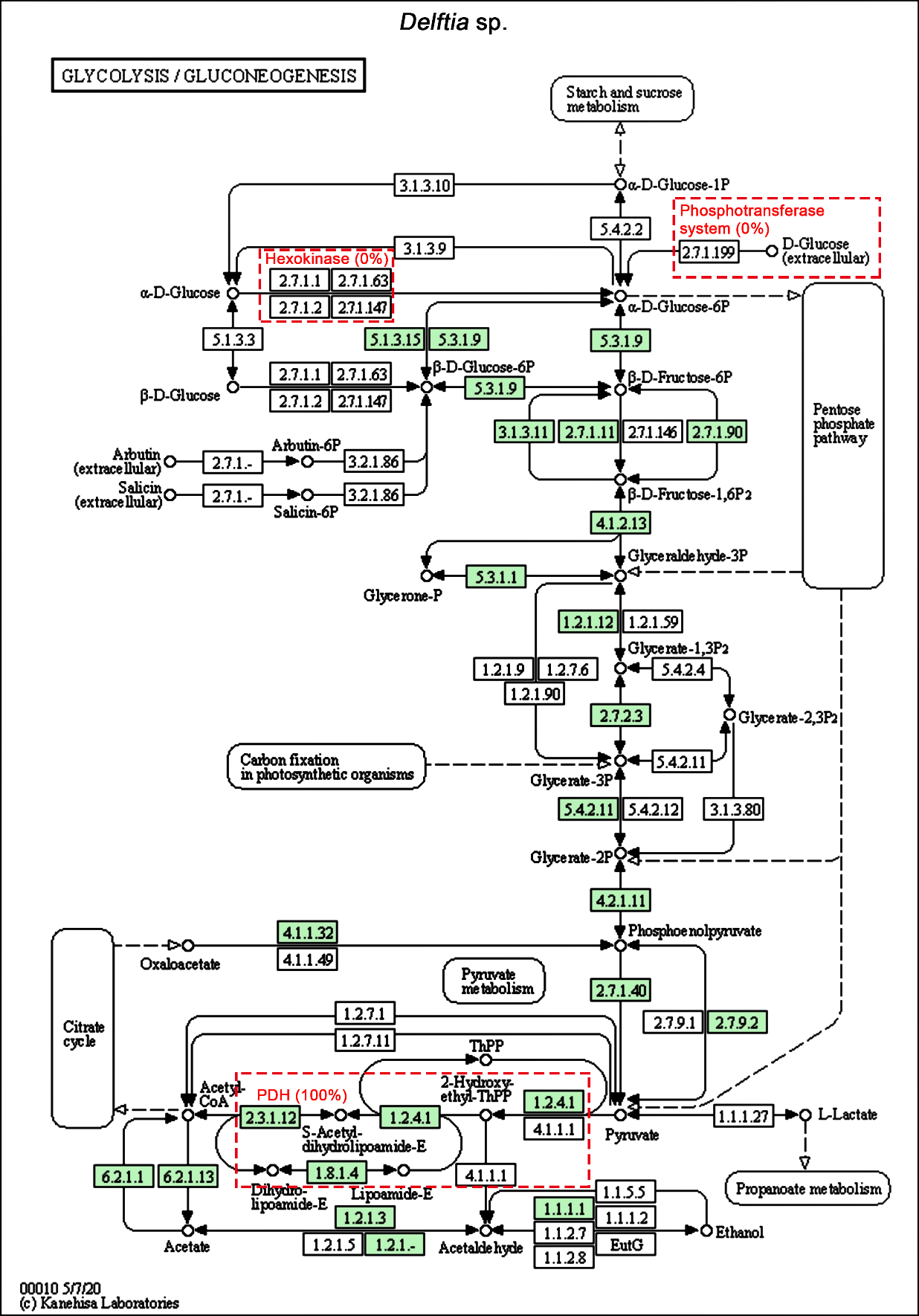

**Figure S21.** The glycolysis and gluconeogenesis metabolic pathways of *Delftia* sp. retrieved from the KEGG pathway database. The percentages indicate the gene proportion within the genus. A green box denotes the presence of the gene in the genus, while a white box indicates the absence of the gene.


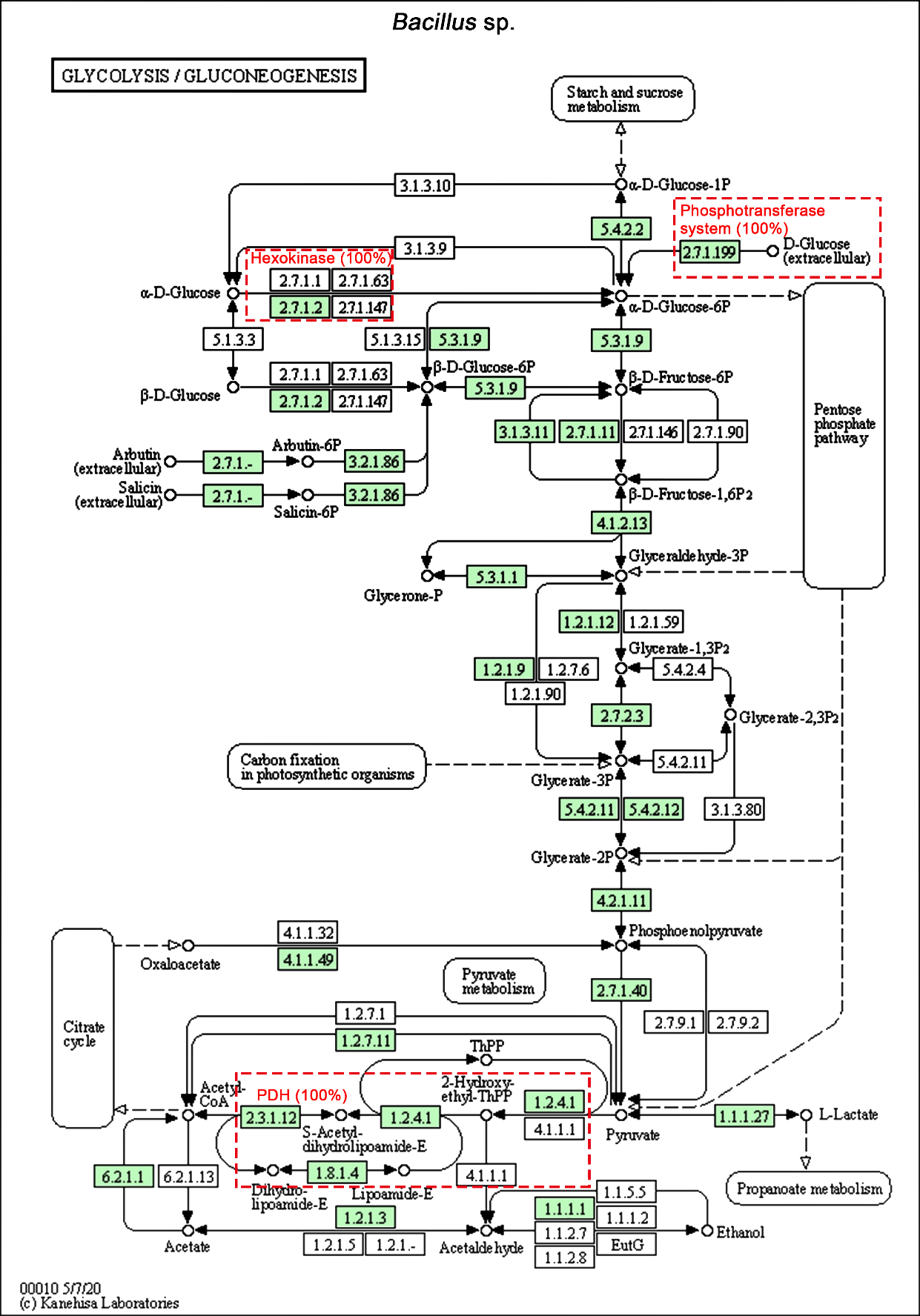


**Figure S22.** The glycolysis and gluconeogenesis metabolic pathways of *Bacillus* sp. retrieved from the KEGG pathway database. The percentages indicate the gene proportion within the genus. A green box denotes the presence of the gene in the genus, while a white box indicates the absence of the gene.

**References**

1. Qiao JT, Li XM, Hu M, Li FB, Young LY, Sun WM, et al. Transcriptional activity of arsenic-reducing bacteria and genes regulated by lactate and biochar during arsenic transformation in flooded paddy soil. Environ Sci Technol. 2018;52:61-70.

2. Xu HJ, Wang XH, Li H, Yao HY, Su JQ, Zhu YG. Biochar impacts soil microbial community composition and nitrogen cycling in an acidic soil planted with rape. Environ Sci Technol. 2014;48:9391-9.

3. Sigler A, He X, Bose M, Cristea A, Liu W, Nam PK, et al. Simultaneous determination of eight urinary metabolites by HPLC-MS/MS for noninvasive assessment of traumatic brain injury. J Am Soc Mass Spectrom. 2020;31:1910-17.

4. Metrani R, Jayaprakasha GK, Patil BS. Optimized method for the quantification of pyruvic acid in onions by microplate reader and confirmation by high resolution mass spectra. Food Chem. 2018;242:451-58.

5. Magdziak Z, Siwulski M, Mleczek M. Characteristics of organic acid profiles in 16 species of wild growing edible mushrooms. J Environ Sci Health B. 2017;52:784-89.

6. Huang K, Xu Y, Packianathan C, Gao F, Chen C, Zhang J, et al. Arsenic methylation by a novel ArsM As(III) S-adenosylmethionine methyltransferase that requires only two conserved cysteine residues. Mol Microbiol. 2018;107:265-76.

7. Dheeman DS, Packianathan C, Pillai JK, Rosen BP. Pathway of human AS3MT arsenic methylation. Chem Res Toxicol. 2014;27:1979-89.

8. Huang K, Chen C, Zhang J, Tang Z, Shen Q, Rosen BP, et al. Efficient arsenic methylation and volatilization mediated by a novel bacterium from an arsenic-contaminated paddy soil. Environ Sci Technol. 2016;50:6389-96.
